# p53 and TAp63 participate in the recombination-dependent pachytene arrest in mouse spermatocytes

**DOI:** 10.1371/journal.pgen.1006845

**Published:** 2017-06-15

**Authors:** Marina Marcet-Ortega, Sarai Pacheco, Ana Martínez-Marchal, Helena Castillo, Elsa Flores, Maria Jasin, Scott Keeney, Ignasi Roig

**Affiliations:** 1 Genome Integrity and Instability Group, Institut de Biotecnologia i Biomedicina, Universitat Autònoma de Barcelona, Cerdanyola del Vallès, Barcelona, Spain; 2 Department of Cell Biology, Physiology and Immunology, Cytology and Histology Unit, Universitat Autònoma de Barcelona, Cerdanyola del Vallès, Barcelona, Spain; 3 Department of Biochemistry and Molecular Biology, Graduate School of Biomedical Sciences, The University of Texas M. D. Anderson Cancer Center, Houston, Texas, United States of America; 4 Developmental Biology Program, Memorial Sloan Kettering Cancer Center, New York, New York, United States of America; 5 Molecular Biology Program, Memorial Sloan Kettering Cancer Center, New York, New York, United States of America; 6 Howard Hughes Medical Institute, Memorial Sloan Kettering Cancer Center, New York, New York, United States of America; Cornell University, UNITED STATES

## Abstract

To protect germ cells from genomic instability, surveillance mechanisms ensure meiosis occurs properly. In mammals, spermatocytes that display recombination defects experience a so-called recombination-dependent arrest at the pachytene stage, which relies on the MRE11 complex—ATM—CHK2 pathway responding to unrepaired DNA double-strand breaks (DSBs). Here, we asked if p53 family members—targets of ATM and CHK2—participate in this arrest. We bred double-mutant mice combining a mutation of a member of the p53 family (p53, TAp63, or p73) with a *Trip13* mutation. *Trip13* deficiency triggers a recombination-dependent response that arrests spermatocytes in pachynema before they have incorporated the testis-specific histone variant H1t into their chromatin. We find that deficiency for either p53 or TAp63, but not p73, allowed spermatocytes to progress further into meiotic prophase despite the presence of numerous unrepaired DSBs. Even so, the double mutant spermatocytes apoptosed at late pachynema because of sex body deficiency; thus p53 and TAp63 are dispensable for arrest caused by sex body defects. These data affirm that recombination-dependent and sex body-deficient arrests occur via genetically separable mechanisms.

## Author summary

Meiosis is a specialized cell division that generates haploid gametes by halving chromosome content through two consecutive rounds of chromosome segregation. At the onset of the first meiotic division, SPO11 protein introduces double-strand breaks (DSBs) throughout the genome. These DSBs are repaired through homologous recombination, which promotes pairing and synapsis of the homologous chromosomes. Some DSBs will become repaired as crossovers, providing a physical connection between the homologous chromosomes which promotes correct chromosome segregation. In fact, recombination defects can lead to formation of aneuploid gametes, one of the major causes of miscarriages and chromosome abnormalities in humans. To protect germ cells from genomic instability and to produce balanced gametes, surveillance mechanisms ensure that meiosis occurs properly. It is known that in the presence of unrepaired DSBs a control mechanism promotes a spermatogenic block at the pachytene stage. Here we describe that, downstream MRE11-ATM-CHK2 pathway, p53 and TAp63 are the effectors responsible for activating recombination-dependent arrest in mouse spermatocytes.

## Introduction

The mammalian p53 family includes p53 [1,2], p63 [3] and p73 [4], which are transcription factors encoded by three highly conserved genes [5,6]. Each member has three major domains: an amino-terminal transactivation (TA) domain, a central DNA binding domain, and a carboxy-terminal oligomerization domain [5,7]. Alternative promoters express two isoforms of p63 and p73. The transactivating isoforms contain the TA domain and the ΔN isoforms lack it [7,8]. Generally, the TA isoforms tend to have tumor suppressor activities, while the ΔN isoforms act as dominant-negative inhibitors that can bind DNA but do not promote transcription [5,7,8]. Splicing variation at the 3′ end of the mRNAs generates additional isoforms [5,8,9].

The interplay between the family members, their isoforms, their differential tissue expression, and their ability to oligomerize yields a high complexity and diverse biological functions including prominent roles in the DNA damage response (DDR) [10–12]. In somatic cells responding to DSBs, the MRE11–RAD50–NBS1 (MRN) complex senses DSBs and activates ATM kinase [13]. ATM then phosphorylates a large set of downstream targets involved in DNA repair, cell cycle progression, and apoptosis, including CHK2 [14,15] and p53 among others [16–18]. Once phosphorylated, p53 is stabilized and mediates cell cycle arrest until DNA damage is repaired [19–21]. If DNA damage persists, p53 levels increase and induce pro-apoptotic genes [22,23]. Similarly to p53 [24], major cellular functions of p63 and p73 include regulating DNA repair, cell cycle progression, and programmed cell death [7,25,26].

One endogenous source of DSBs occurs early in the first meiotic prophase, when SPO11 protein introduces numerous DSBs that are subsequently repaired by homologous recombination to promote chromosome pairing and synapsis [27,28]. Since errors at this point can cause genomic instability and introduce germ line mutations, mechanisms exist to detect defects in recombination and other processes during prophase, and if necessary delay cell cycle progression and/or promote programmed cell death [29–31]. These mechanisms are generically referred to as the pachytene checkpoint, and in male mouse meiosis can be divided into two main arrest pathways. One responds to defective repair of DSBs (referred to as recombination-dependent arrest) [29,32]. The other responds to failure in transcriptional silencing of the non-homologous portions of the sex chromosomes (sex body-deficient arrest) [33,34]. Genetic pathways responsible for these quality control systems remain incompletely understood.

**Fig 1:**
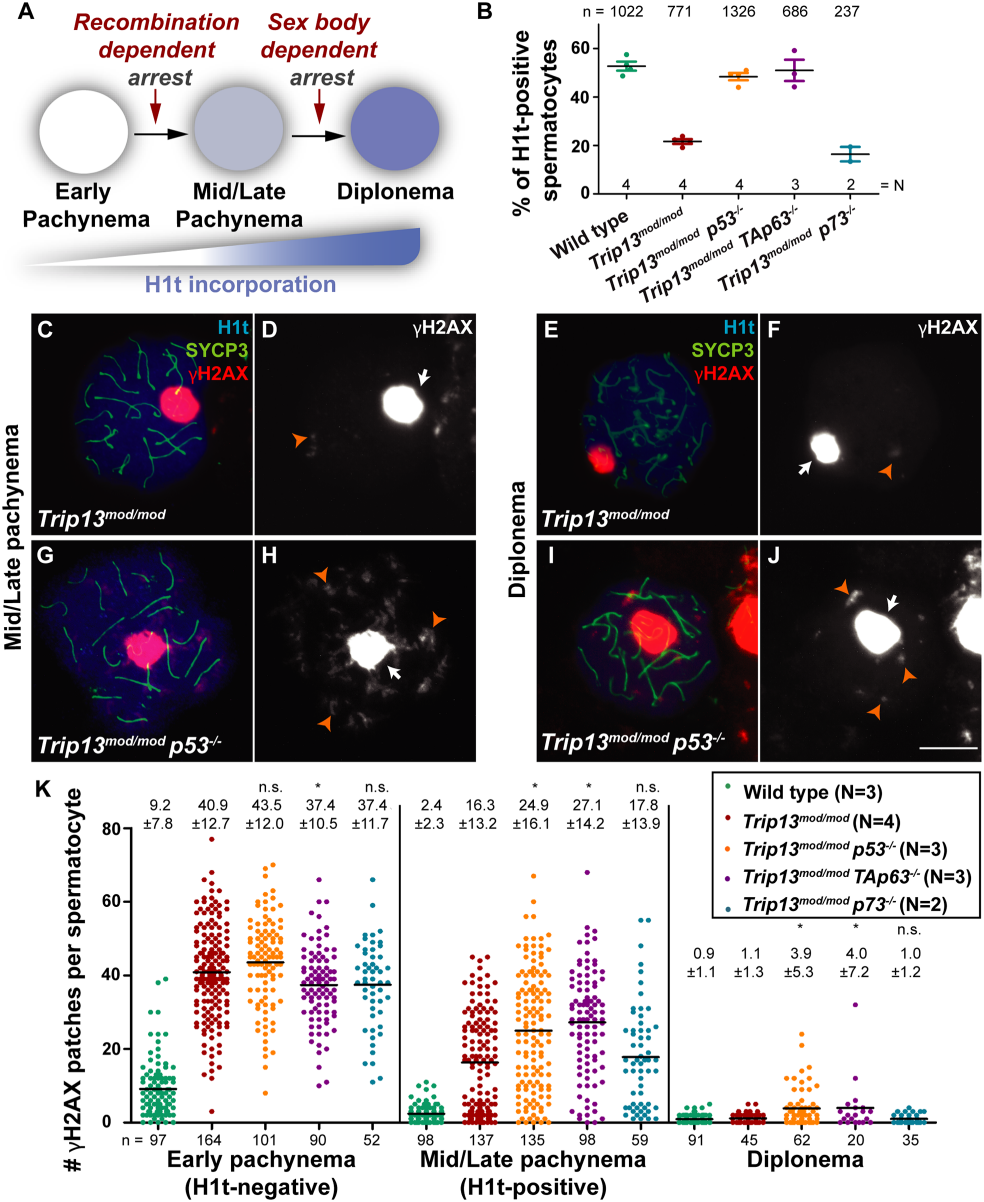
Absence of p53 or TAp63 allows TRIP13-deficient spermatocytes to accumulate H1t despite having multiple unrepaired DSBs. (A) Schematic representation of arrest events and H1t incorporation from early pachynema to diplonema. (B) Graph showing the average percentage of spermatocytes that have incorporated H1t for each genotype and error bars represent standard deviation (SD). (C-J) Spread chromosomes from representative spermatocytes of the indicated genotypes and stages, stained for the axial element protein SYCP3 (green), H1t (blue), and γH2AX (red). The large, bright blobs of γH2AX staining are sex bodies (arrows); the smaller γH2AX patches reflect unrepaired DSBs (orange arrowheads). Scale bar in J represents 10 μm and applies to all panels. (K) Quantification of the number of γH2AX patches per spermatocyte. Horizontal lines represent means. Means (± SD) are indicated above the graph. Above the means, (n.s.) indicates not significant and (*) indicates significantly different relative to *Trip13*^*mod/mod*^. (Statistical tests and p values are stated in main text). The total number of cells studied (n) and the number of analyzed animals per genotype (N) are shown in each graph.

These arrest mechanisms can be distinguished cytologically using the incorporation of the testis-specific histone variant H1t [29], which in wild-type mice accumulates on chromatin from mid-pachynema onwards (Fig 1A) [35]. Mutant spermatocytes with persistent unrepaired DSBs (e.g., in *Dmc1*^*-/-*^) arrest at pachynema before expressing H1t, whereas H1t accumulates in mutant spermatocytes that do not form DSBs but have sex body defects (e.g., *Spo11*^*-/-*^) [29,36]. This difference suggests that defects in DSB repair provoke arrest at an earlier substage of pachynema than is seen for defects in sex body formation. Nonetheless, both recombination-dependent arrest and sex body-deficient arrest cause apoptosis at stage IV of the seminiferous epithelial cycle, corresponding to mid-pachynema in wild type [29,36].

Recent studies have identified some of the proteins involved in recombination-dependent arrest in mouse spermatocytes and oocytes, using a hypomorphic gene-trap mutant allele of the *Trip13* gene, *Trip13*^*mod/mod*^ (also known *Trip13*^*Gt/Gt*^) [36,37]. TRIP13 is an AAA+ ATPase that is required for the completion of meiotic recombination, but is dispensable for homologous chromosome synapsis [38,39]. In spermatocytes, recombination-dependent arrest in response to *Trip13* mutation depends on components of the somatic DDR, namely, the MRN complex, ATM, and CHK2 [36]. Most *Trip13*^*mod/mod*^ spermatocytes arrest before incorporating H1t and display hallmarks of unrepaired DSBs, but removing or reducing ATM activity or eliminating CHK2 allows *Trip13*-mutant spermatocytes to progress further and incorporate H1t [36].

CHK2 is also necessary for eliminating *Trip13*^*mod/mod*^ oocytes with persistent unrepaired DSBs, via activation of p53 and TAp63 [37]. Moreover, TAp63 is necessary for elimination of irradiated oocytes [40]. These findings in oocytes raise the possibility that p53 family members may also participate in similar arrest responses in spermatocytes. Notably, all p53 family members, p53, p63 and p73, are expressed in mammalian spermatocytes [41,42]. Moreover, this hypothesis is consistent with findings that p53 is activated in mouse spermatocytes in response to SPO11-generated DSBs [43] and is involved in arresting *Arf*^*-/-*^ spermatocytes, which present high levels of γH2AX at pachynema [44]. Hence, we investigated whether p53 family members mediate recombination-dependent arrest in mouse spermatocytes.

## Results

### p53 and TAp63, but not p73, promote recombination-dependent arrest in mouse spermatocytes

If a p53 family member is required for recombination-dependent arrest, then removing that protein should allow *Trip13*-deficient spermatocytes to progress further into meiotic prophase. We tested this prediction by combining p53 family member mutations with *Trip13* mutation (*Trip13*^*mod/mod*^ *p53*^*-/-*^, *Trip13*^*mod/mod*^ *TAp63*^*-/-*^, and *Trip13*^*mod/mod*^ *p73*^*-/-*^) and analyzing the mutants’ meiotic phenotypes. For p63, we used a mutation that specifically eliminates its TA forms [45] because *p63*-null mice die perinatally [46].

First, as we previously demonstrated for *Trip13*^*mod/mod*^ *Chk2*^*-/-*^ mutants [36], we asked if the p53-family double mutants yielded more cells expressing H1t compared to the *Trip13*^*mod/mod*^ single mutant. To do this, we stained squashed spermatocyte preparations for SYCP3 (a component of the axial element of the synaptonemal complex) [47] and H1t. As expected [36], only 21.6% of spermatocytes were H1t-positive in *Trip13*^*mod/mod*^ single mutants, compared with 52.7% in wild type (Fig 1B). The reduction in *Trip13*^*mod/mod*^ single mutants is a consequence of recombination-dependent arrest, with most of the H1t-positive cells representing a relatively recombination-proficient subset of cells (“escapers”) attributable to the partial penetrance of the *Trip13* hypomorphic recombination defect [36,38,39]. In contrast, we recovered a significantly greater fraction of H1t-positive spermatocytes in the *p53* and *TAp63* double mutants compared with the *Trip13*^*mod/mod*^ single mutant (48.4% of *Trip13*^*mod/mod*^ *p53*^*-/-*^ and 51.0% of *Trip13*^*mod/mod*^ *TAp63*^*-/-*^ spermatocytes; p≤0.001, one-way ANOVA test for both comparisons). The *p73* double mutant had 16.4% of H1t-positive spermatocytes, comparable to *Trip13*^*mod/mod*^ (p>0.05, one-way ANOVA test, Fig 1B). These results suggest that in the absence of p53 or TAp63, but not p73, *Trip13* mutant spermatocytes are able to bypass the recombination-dependent arrest.

If this interpretation is correct, we would expect that p53 or TAp63 deficiency allows *Trip13*-mutant spermatocytes to progress despite the presence of unrepaired DSBs. We therefore stained spermatocyte spreads for γH2AX, the phosphorylated form of the histone variant H2AX that marks DSBs [48], and counted the number of γH2AX patches present in each cell (Fig 1, S1 and S2 Figs). Previously, we showed that *Trip13*^*mod/mod*^ spermatocytes accumulate numerous patches of γH2AX on fully synapsed chromosomes in early pachynema, indicative of persistent unrepaired DSBs [36,38] (Fig 1K, left graph). Many of these patches persisted in H1t-positive cells, but two subpopulations of the mid/late pachytene *Trip13*^*mod/mod*^ spermatocytes were apparent: one with low numbers of γH2AX patches, similar to wild type, and another retaining numerous patches (Fig 1K, middle graph). However, those cells that reached diplonema did so with normal (very low) numbers of γH2AX patches (Fig 1K, right graph). As previously argued [36], we infer that stochastic cell-to-cell differences in the number of unrepaired DSBs translate into different arrest responses: cells with numerous DSBs experience recombination-dependent arrest early in pachynema (H1t-negative), cells with an intermediate number progress to an H1t-positive stage before apoptosing (possibly because of the DSBs, sex body defects, or both; see below), and the most repair-proficient cells progress still further to diplonema.

Before the point of recombination-dependent arrest (early pachynema, H1t-negative cells), the numbers of γH2AX patches in the *p53* and *p73* double mutants were not statistically significantly different from the *Trip13*^*mod/mod*^ single mutant (p = 0.091 and p = 0.086, t test, respectively) (Fig 1K, left graph). γH2AX patches were slightly decreased in the *TAp63* double mutant (p = 0.027, t test), but still much higher than in wild type. We infer that the absence of the p53 family members does not greatly ameliorate the DSB repair defect caused by TRIP13 deficiency.

For the purpose of assessing arrest status of cells with unrepaired DSBs, the key stages are mid/late pachynema (H1t-positive) and diplonema. As predicted for defects in recombination-dependent arrest, the *p53* and *TAp63* double mutants had a higher average number of γH2AX patches in H1t-positive cells than was observed in the *Trip13*^*mod/mod*^ single mutant (p≤0.0001, t test; Fig 1C, 1D, 1G, 1H and 1K and S1A and S1B Fig). This increase reflects less of an enrichment for the γH2AX-low subpopulation plus an increased occurrence of cells with γH2AX patches at or above the high end of the range seen in the single mutant (Fig 1K, middle graph). Both double mutants also displayed more γH2AX patches in diplotene cells (p = 0.00001 for *p53*, and p = 0.00029 for *TAp63*, negative binomial regression; Fig 1E, 1F, 1I, 1J and 1K (right graph) and S1C and S1D Fig). Similar results were obtained when RAD51 was used as a marker for recombination events: the *p53* double mutant had similar numbers of RAD51 foci as the *Trip13*^*mod/mod*^ single mutant at early pachynema, but had elevated numbers of RAD51 foci at mid/late pachynema and diplonema (S3 Fig). Overall, these data indicate that spermatocytes with substantial numbers of unrepaired DSBs are more likely to progress to the H1t-positive stage and beyond if p53 or TAp63 are missing, similar to our previous findings for absence of CHK2 [36]. In contrast, the *p73* double mutant was similar to the *Trip13*^*mod/mod*^ single mutant, both in mid/late pachynema (p = 0.49, t test; Fig 1K, middle graph, and S1E and S1F Fig) and in diplonema (p = 0.65, negative binomial regression; Fig 1K, right graph, and S1G and S1H Fig). This finding corroborates the conclusion that, even though p73 is expressed in mouse spermatocytes [42], it is dispensable for recombination-dependent arrest.

**Fig 2:**
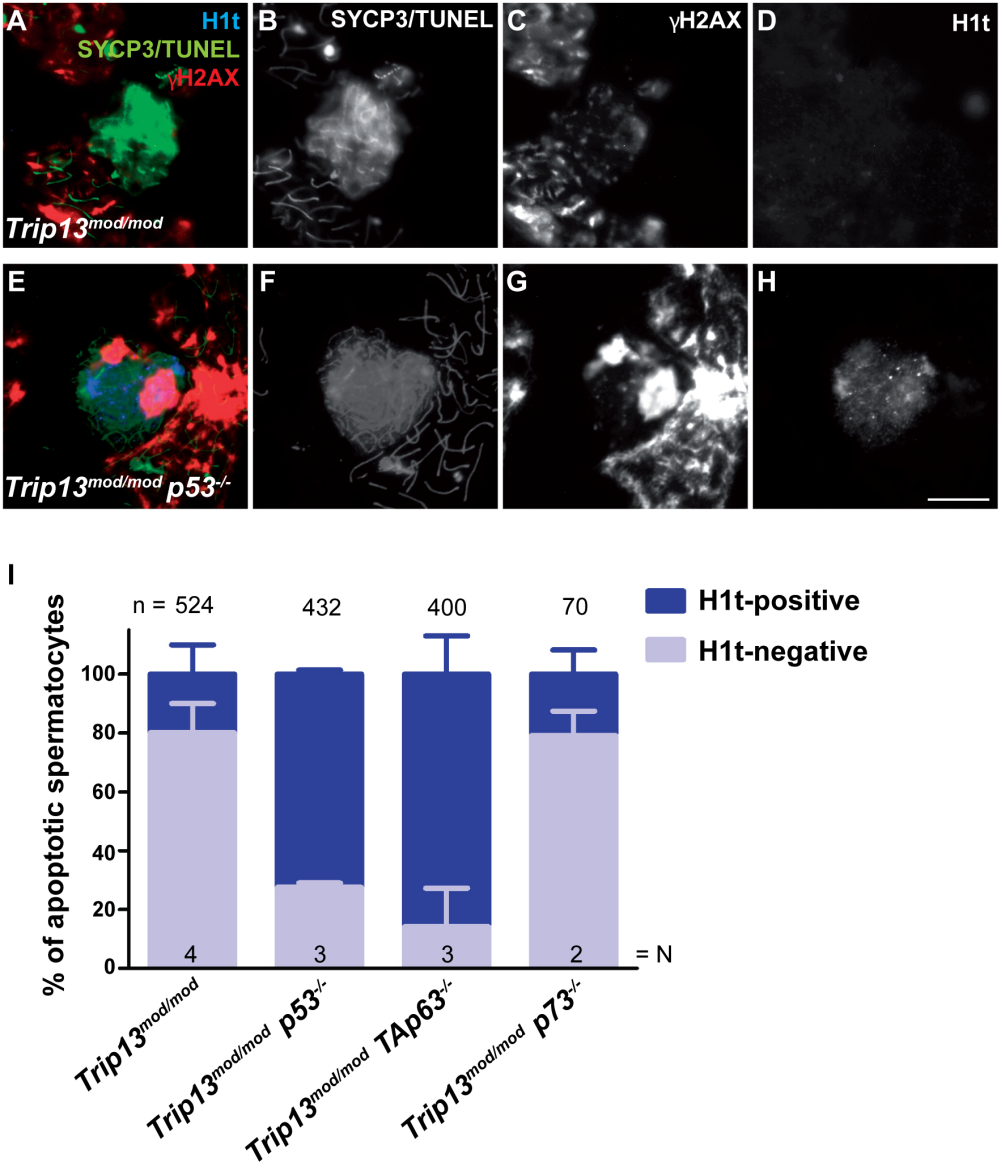
Apoptosis tends to occur at a later (H1t-positive) stage in TRIP13-deficient spermatocytes lacking p53 or TAp63. (A-H) Representative apoptotic (TUNEL-positive) pachytene-stage spermatocytes stained for SYCP3 and TUNEL (both in green), H1t (blue), and γH2AX (red). Note the presence of multiple γH2AX patches and abnormal sex body in the apoptotic, H1t-positive spermatocyte from the *Trip13*^*mod/mod*^ *p53*^*-/-*^ mutant. Scale bar in H represents 10 μm and applies to all panels. (I) Percentage of apoptotic spermatocytes that are H1t-negative or H1t-positive. Error bars represent SD. The total number of cells studied (n) and the number of analyzed animals per genotype (N) are shown. Note that error bars from *Trip13*^*mod/mod*^ *p53*^*-/-*^ mutant are almost too small to be seen.

To determine if absence of p53 or TAp63 affects apoptosis of TRIP13-deficient spermatocytes, we performed TUNEL staining of double mutant spermatocytes (Fig 2 and S4 Fig). As expected from prior results [36], most (80.1%) TUNEL-positive spermatocytes were H1t-negative in the *Trip13*^*mod/mod*^ single mutant (Fig 2A–2D and 2I). The *p73* double mutant was similar, with 79.2% of apoptotic spermatocytes being H1t-negative (p = 0.92 compared to *Trip13*^*mod/mod*^, one-way ANOVA test; Fig 2I and S4E–S4H Fig). In striking contrast, the majority of apoptotic spermatocytes in the *p53* and *TAp63* double mutants were H1t-positive (only 27.6% H1t-negative for *p53*, p≤0.0001; and 14.2% for *TAp63*, p = 0.001; one-way ANOVA test) (Fig 2E–2H and 2I and S4A–S4D Fig). These results indicate that both p53 and TAp63, but not p73, promote the elimination of most spermatocytes with numerous unrepaired DSBs in early pachynema.

### Mutually independent up-regulation of p53 and p63 protein levels in *Trip13*^*mod/mod*^ spermatocytes

**Fig 3:**
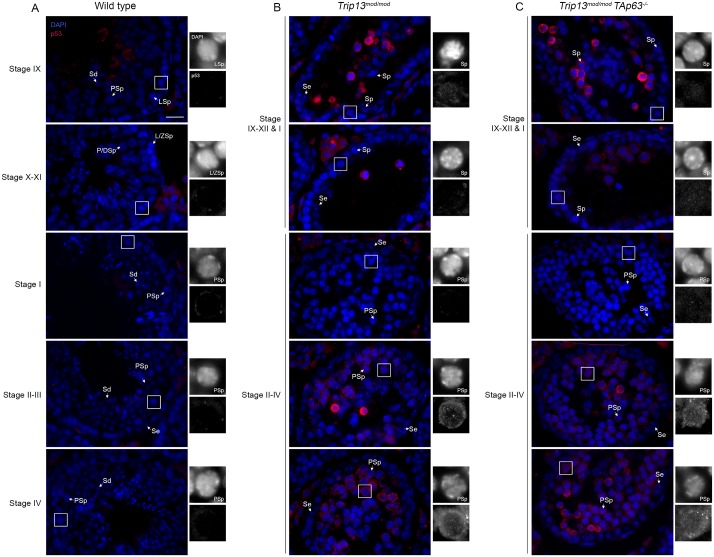
p53 is up-regulated in TRIP13-deficient pachytene spermatocytes. Representative testis sections from wild type (A), *Trip13*^*mod/mod*^ (B), and *Trip13*^*mod/mod*^ *TAp63*^*-/-*^ (C) stained with DAPI and for p53 are shown. Cross sections of tubules of different epithelial stages are shown to illustrate staining patterns during progression of spermatocytes from leptonema to pachynema. Insets show marked spermatocytes from different stages. Note that the presence of all spermatogenic cells allows for the precise staging of the tubules in wild type samples. However, the absence of most secondary spermatocytes and spermatids impedes an accurate staging of *Trip13* mutant tubule sections. Nonetheless, tubules in which spermatocytes make up the outermost layer of cells can be clearly assigned to stages IX-XII or I. Such tubules contain in the most exterior layer of cells spermatocytes from leptonema to early pachynema. Labels on the panels stand for: Se: Sertoli cell, LSp: leptotene spermatocyte, L/ZSp: leptotene/zygotene spermatocyte, P/DSp: pachytene/diplotene spermatocyte, Sp: spermatocyte, Sd: spermatid. Scale bar represents 20 μm and applies to all panels.

**Fig 4:**
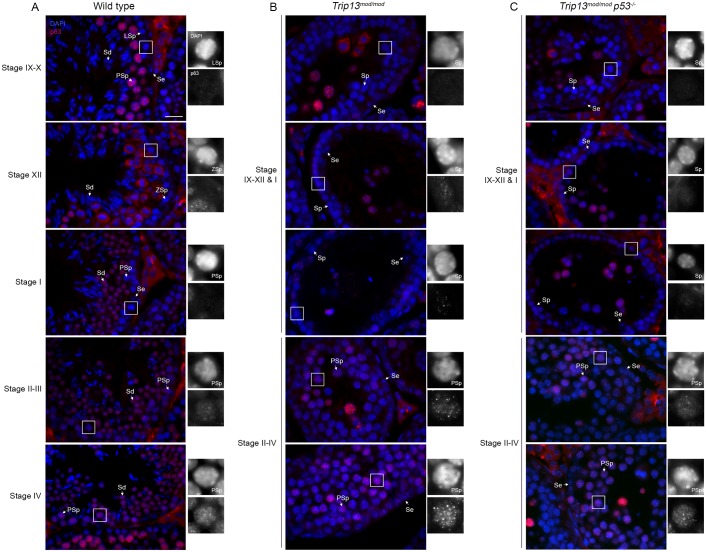
Premature p63 up-regulation in response to TRIP13 deficiency. Representative testis sections from wild type (A), *Trip13*^*mod/mod*^ (B), and *Trip13*^*mod/mod*^ *p53*^*-/-*^ (C) stained with DAPI and for p63 are shown, organized as for Fig 3. Note that in *Trip13* mutants (B-C), p63 signal appears in the outermost layer of cells of tubules that are at stage IX-XII or I, which corresponds to leptotene to early pachytene stage spermatocytes (sp). In wild-type tubules, early pachytene cells in stage I tubules show no staining for p63, which only appears in pachtyene spermatocytes (Psp) in stage II-III tubules onwards. Thus, p63 expression is prematurely upregulated in *Trip13* mutant testis. Labels on the panels stand for: Se: Sertoli cell, LSp: leptotene spermatocyte, L/ZSp: leptotene/zygotene spermatocyte, P/DSp: pachytene/diplotene spermatocyte, Sp: spermatocyte, Sd: spermatid. Scale bar represents 20 μm and applies to all panels. Scale bar represents 20 μm and applies to all panels.

To gain insight into how p53 and TAp63 may collaborate to promote recombination-dependent arrest, we analyzed the expression of p53 and p63 proteins in *Trip13*^*mod/mod*^ single mutants and in the double mutants by immunofluorescent staining of testis sections (Figs 3 and 4). Each cross section of a seminiferous tubule can be classified as one of twelve epithelial stages (I to XII) on the basis of the developmental stages of germ cells present in the section [49,50]. Epithelial staging can also be deduced for mutants that experience spermatogenic arrest, although precise stage assignments are not always possible [50].

Similar to a recent report [51], a p53 antibody stained only occasional cells within testis sections from wild type, with most germ cells showing little or no detectable staining at any stage of spermatogenesis (Fig 3A). In the *Trip13*^*mod/mod*^ single mutant, spermatocytes judged to be in leptonema through zygonema similarly had little if any detectable signal, but around one third of tubules at stages II through IV contained numerous pachtyene spermatocytes with strong anti-p53 antibody staining (Fig 3B). This signal was highest in the cytoplasm but was also apparent within nuclei (insets in Fig 3B). Particularly brightly stained cells were also apparent in more luminal positions in tubules of stages IX through XII or stage I; these are likely to be the escapers that have progressed further through meiotic prophase. The *TAp63* double mutant showed p53 staining patterns indistinguishable from the *Trip13*^*mod/mod*^ single mutant (Fig 3C). Thus, p53 protein levels are up-regulated during pachynema in response to defects caused by TRIP13 deficiency, and this up-regulation is independent of TAp63.

In wild-type spermatocytes, the p63 antibody stained spermatocyte nuclei in a focal pattern, beginning faintly in early pachynema (in stage II through IV tubules) and increasing in intensity in later pachynema (e.g., in stage IX–X) (Fig 4A). The signal remained bright but was largely excluded from chromatin in metaphase I spermatocytes (stage XII) and was again bright on chromatin in round spermatids (Fig 4A). These patterns match precisely with a prior report [42]. In the *Trip13*^*mod/mod*^ single mutant, p63 staining became prominent at earlier stages, with a subset of early prophase cells showing detectable p63 foci (i.e., at or before the beginning of pachynema; stages IX through XII or stage I) and with similar staining as in wild type in pachytene spermatocytes in stage II–IV tubules (Fig 4B). Presumptive escapers also stained brightly (e.g., in stages IX through XII). The *Trip13 p53* double mutant showed p63 staining patterns equivalent from the *Trip13*^*mod/mod*^ single mutant (Fig 4C). These findings indicate that p63 levels are up-regulated prematurely in the absence of TRIP13, and this up-regulation is independent of p53 status.

These results are consistent with both p53 and TAp63 being involved in responses to unrepaired DSBs in the *Trip13*^*mod/mod*^ mutant, and thus being involved in implementing recombination-dependent spermatocyte arrest. A simple explanation for why both proteins are required for arrest could have been that they were mutually dependent for their expression. However, the independence of their up-regulation in the *Trip13*^*mod/mod*^ mutant argues strongly against this possibility. Possible models to explain why up-regulation of one without the other is not sufficient to trigger arrest are provided in the Discussion.

### *Trip13*^*mod/mod*^ spermatocytes lacking *p53* or *TAp63* arrest at epithelial stage IV because of defective sex body formation

Most of the lengths of the X and Y chromosomes are non-homologous and thus remain unsynapsed during meiotic prophase. The unsynapsed sex chromosomes undergo transcriptional silencing, termed meiotic sex chromosome inactivation (MSCI), which results in formation of the heterochromatic sex body [34]. MSCI is crucial for meiotic progression, since expression of sex chromosome genes during pachynema is deleterious for spermatocytes [33]. We previously reported that *Trip13*^*mod/mod*^ *Chk2*^*-/-*^ spermatocytes, although escaping recombination-dependent arrest, nonetheless arrested at epithelial stage IV due to the defects in sex body function that are another consequence of TRIP13 deficiency [36]. Because removing *p53* or *TAp63* allowed *Trip13* mutants to progress further into meiosis but did not prevent eventual apoptosis of spermatocytes (Fig 2), we characterized this cell death in more detail.

**Fig 5:**
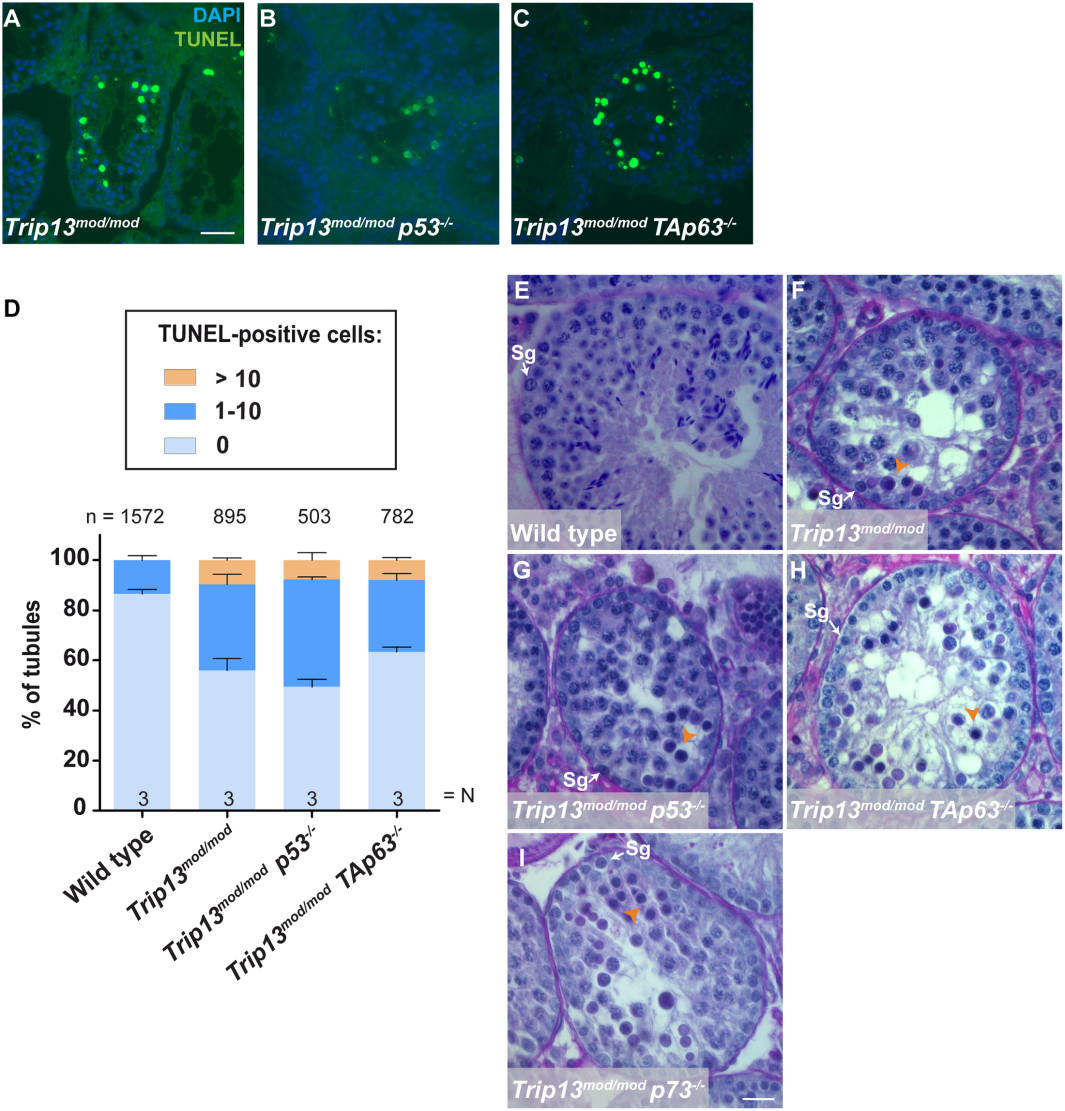
Absence of p53 family members does not alleviate spermatocyte apoptosis at epithelial stage IV in TRIP13-deficient mice. (A–C) Representative tubules are shown from testis sections of the indicated genotypes stained by TUNEL to detect apoptotic spermatocytes (green). Scale bar in A represents 40 μm and applies to panels A–C. (D) Quantification of the percentage of tubules with 0, 1–10, or more than 10 TUNEL-positive cells. The total number of tubules studied (n) and the number of analyzed animals per genotype (N) are shown. (E–I) Representative tubule sections from adult testes stained with PAS-Hematoxylin. Scale bar in I represents 20 μm and applies to panels E–I. (E) Wild-type seminiferous tubule at stage IV containing spermatogonia, spermatocytes and spermatids. (F–I) *Trip13*^*mod/mod*^ single mutant and p53 family member double mutant tubule sections showing condensed spermatocytes (apoptotic, orange arrowheads). These tubules can be assigned to stage IV on the basis of the presence of both intermediate (In) and B-type spermatogonia (Sg) along with a single layer of spermatocytes, which are a mix of apoptotic and non-apoptotic pachytene cells [50].

TUNEL staining on histological sections (Fig 5A–5C) demonstrated that the occurrence of apoptosis in the *p53* and *TAp63* double mutants was similar to the *Trip13*^*mod/mod*^ single mutant (Fig 5D). Importantly, by histological analysis, *p53* and *TAp63* double mutants both showed arrest at epithelial stage IV (Fig 5E–5H), as do *Trip13*^*mod/mod*^ single mutants and *Trip13*^*mod/mod*^ *Chk2*^*-/-*^ double mutants [36]. The *p73* double mutant also displayed arrest at stage IV (Fig 5I).

**Fig 6:**
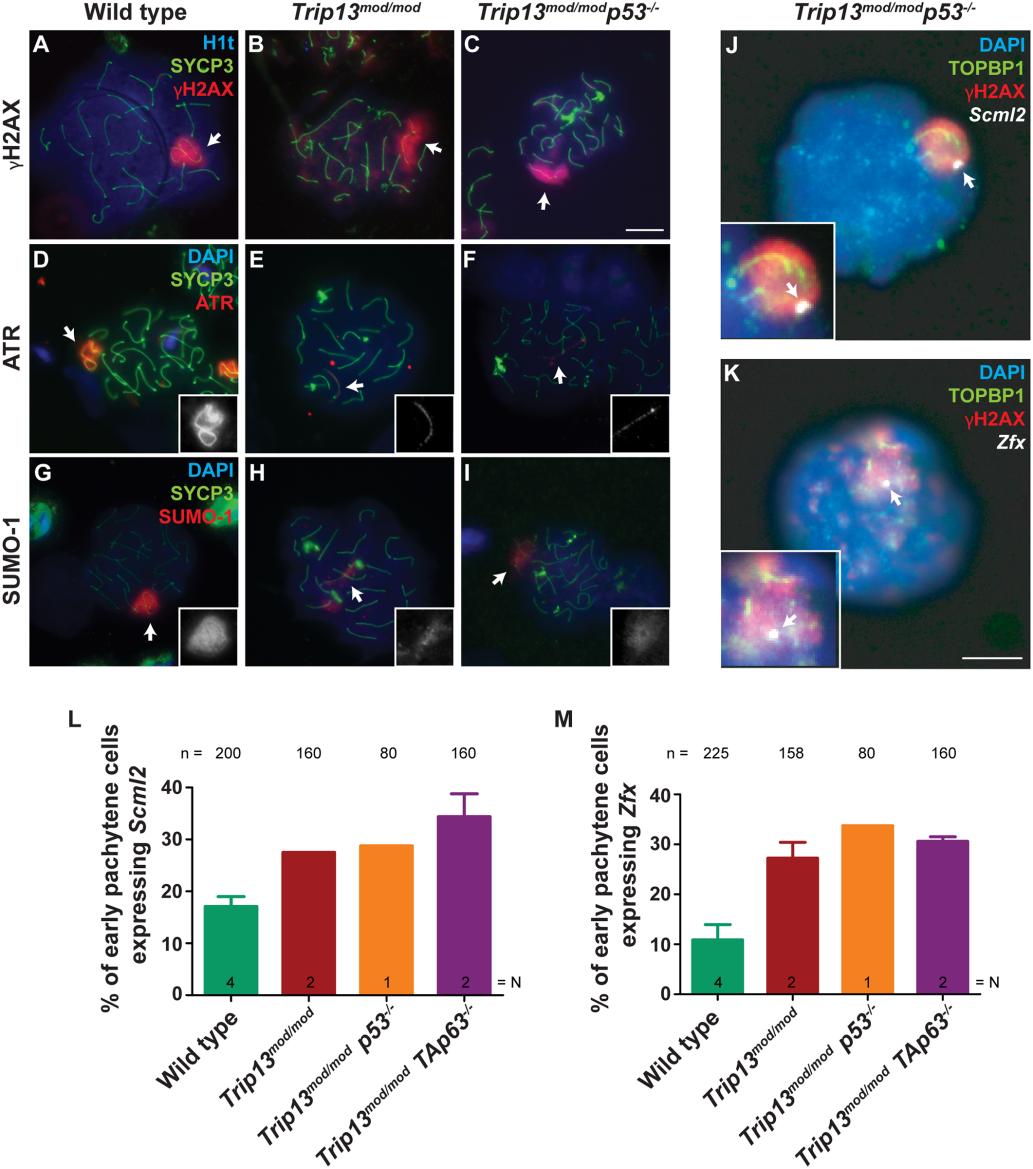
Sex body defects and MSCI failure in TRIP13-deficient cells lacking p53 or TAp63. (A–C) Representative H1t-positive pachytene spermatocytes of the indicated genotypes stained for SYCP3 (green), γH2AX (red), and H1t (blue). (D–I) Representative pachytene spermatocytes of the indicated genotypes stained for SYCP3 (green), DAPI (blue), and either ATR (D–F) or SUMO-1 (G–I) (red). Scale bar in C represents 10 μm and applies for panels A–I. White arrows indicate sex chromosomes. (J,K) RNA-FISH performed on *Trip13*^*mod/mod*^ *p53*^*-/-*^ spermatocytes. Images display *Scml2* or *Zfx* RNA-FISH signal (white arrows), DAPI (blue), and immunostaining for TOPBP1 (green) and γH2AX (red). Insets show zoomed images of the sex bodies. Scale bar on K represents 10 μm and applies to panels J-K. (L,M) Graphs showing percentage of early pachytene spermatocytes expressing *Scml2* (L) or *Zfx* (M) RNA-FISH signal. Wild type and *Trip13*^*mod/mod*^ data are from ref. [36]. Error bars represent SD.

To corroborate that stage IV arrest of *p53* and *TAp63* double mutant spermatocytes can be linked to MSCI failure, we analyzed the morphology and functionality of the sex body. First, we stained spermatocyte spreads for several sex body markers: γH2AX, ATR (the kinase principally responsible for H2AX phosphorylation in the sex body [52]), and SUMO-1 (which accumulates on the X and Y chromatin at pachynema in an ATR-dependent manner [52]) (see Fig 6A–6I for representative images and S1 Table for quantification). For each marker, the *p53* and *TAp63* double mutants displayed sex body abnormalities that were qualitatively and quantitatively indistinguishable from the *Trip13*^*mod/mod*^ single mutant. Specifically, we found abnormally elongated sex bodies in approximately two-thirds of pachytene spermatocytes (compare sex bodies in Fig 6B and 6C and S5A Fig to the round and intensely stained sex body in Fig 6A). Whereas ATR in wild-type pachynema usually covered the unsynapsed X and Y chromosome axes and expanded over their chromatin (Fig 6D), less than 20% of pachytene cells from double mutants or the *Trip13*^*mod/mod*^ single mutant displayed this chromatin-expanded pattern (S1 Table). Instead most cells showed a more discontinuous localization of ATR along the X and Y axes (Fig 6E and 6F and S5B Fig) as well as only modest SUMO-1 staining (Fig 6H and 6I and S5C Fig), unlike the intense signal typical of sex bodies in wild type (Fig 6G). These results confirm that *Trip13* mutants have defects in sex body formation, consistent with previous results [36], and demonstrate that these defects are not ameliorated by absence of either p53 or TAp63.

Next, we assessed MSCI by performing RNA fluorescence *in situ* hybridization (RNA-FISH) analysis of the X-linked genes *Scml2* (located next to the pseudoautosomal region) and *Zfx* (more interstitially located) [53]. Both genes are normally silenced by MSCI throughout pachynema. To identify early pachytene cells, slides were also immunostained for TOPBP1 (DNA topoisomerase 2 binding-protein 1), which associates with the unsynapsed sex chromosomes at pachynema [54]. Similar to our prior findings for the *Trip13*^*mod/mod*^ single mutant [36], a greater fraction of double mutant spermatocytes expressed one or the other X-linked gene than in wild type (Fig 6J–6M and S5D–S5I Fig; further quantification in S2 Table). These results match what we previously observed for *Trip13*^*mod/mod*^ *Chk2*^*-/-*^ mutants [36]. Thus, alleviating recombination-dependent arrest in TRIP13-deficient spermatocytes by removing CHK2, p53, or TAp63 does not overcome the separate pachytene arrest pathway tied to MSCI failure.

**Fig 7:**
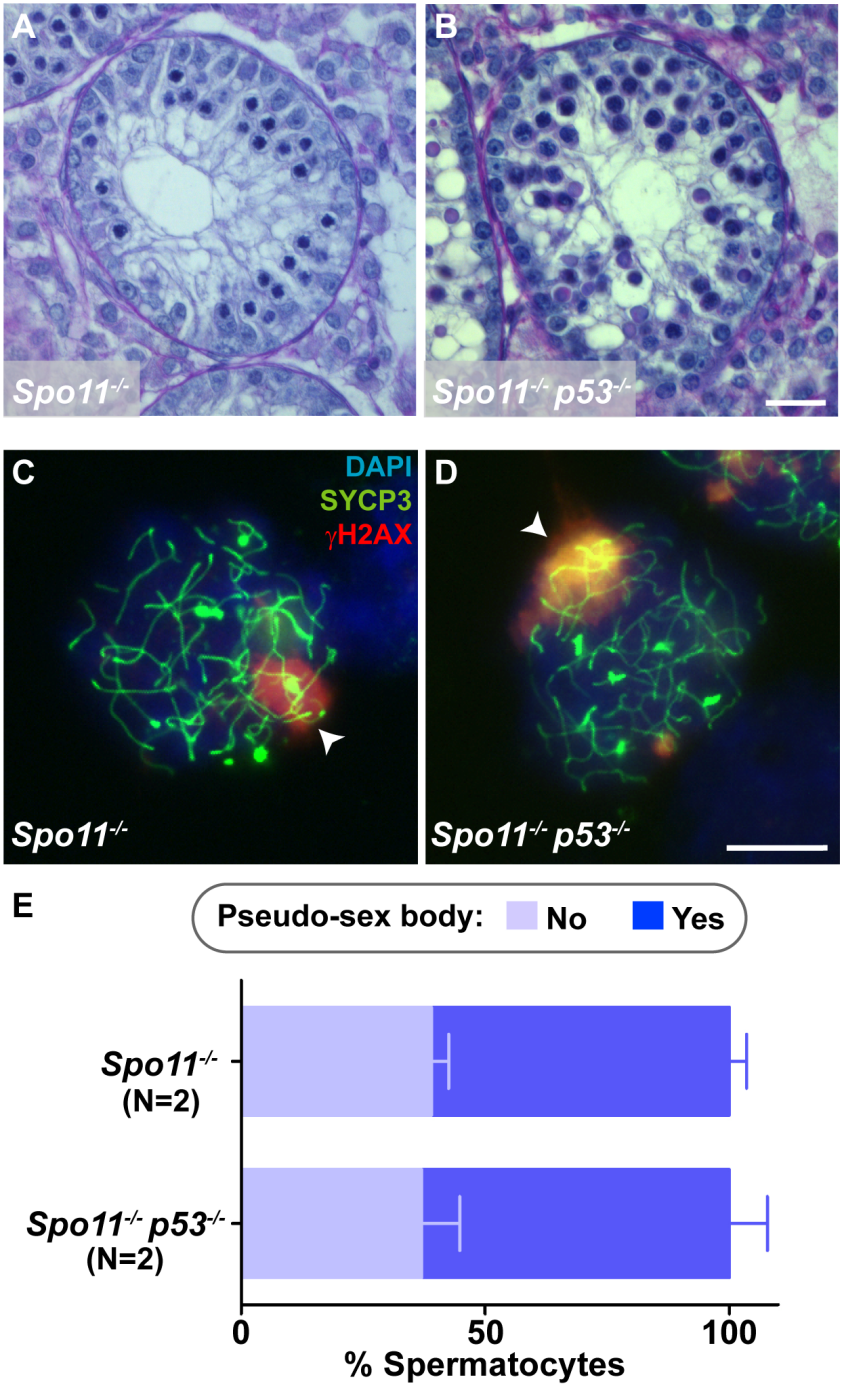
Mutation of *p53* does not rescue *Spo11*^*-/-*^ meiotic arrest. (A,B) Representative tubule sections from adult testes stained with PAS-Hematoxylin. Scale bar in B represents 20 μm and applies to panels A and B. (C,D) Spermatocytes stained for SYCP3 (green), γH2AX (red), and DAPI (blue). Note the presence of a pseudo-sex body in both cells (arrowheads). Scale bar in D represents 10 μm and applies to panels C and D. (E) Graph displaying the mean percentage of *Spo11*^*-/-*^ and *Spo11*^*-/-*^*p53*^*-/-*^ spermatocytes containing a pseudo-sex body. Error bars represent SD. N shows the number of mice analyzed.

To further confirm that p53 family members are dispensable for sex body-deficient arrest, we asked if *p53* or *TAp63* deficiency can ameliorate the arrest caused by absence of *Spo11*. Because they lack DSBs, *Spo11*^*-/-*^ spermatocytes display synapsis defects that lead to failure to form a proper sex body, thus allowing expression of sex chromosome genes and activation of the sex body-deficient arrest [55,56]. We generated *Spo11*^*-/-*^ *p53*^*-/-*^ and *Spo11*^*-/-*^ *TAp63*^*-/-*^ double mutants and analyzed testis weights, which can distinguish between mutants arresting at different meiotic stages. As predicted if p53 or TAp63 are not required for sex body-deficient arrest, testis weights were indistinguishable for *Spo11*^*-/-*^ *p53*^*-/-*^ or *Spo11*^*-/-*^ *TAp63*^*-/-*^ double mutants and *Spo11*^*-/-*^ single mutants (p = 0.22, t test) (S6 Fig). Furthermore, histological analysis of double mutant testis sections revealed the existence of stage IV arrest in *Spo11*^*-/-*^ *p53*^*-/-*^, similarly to what is found in *Spo11*^*-/-*^ mice (Fig 7A and 7B).

We further analyzed meiotic prophase progression cytologically in these two mutants. *Spo11* mutant spermatocytes fail to complete synapsis, thus the most advanced spermatocyte that can be observed is a zygotene-like cell [55,56]. We showed previously that the *Spo11*^*-/-*^ spermatocytes that apoptose are a subset of these zygotene-like cells that displayed a pseudo sex body [36], i.e., such spermatocytes are the most advanced germ cells present in *Spo11* mutant testis. Therefore, to determine if *p53* mutation affected the ability of *Spo11* mutant spermatocytes to progress, we analyzed the presence of zygotene-like spermatocytes containing a pseudo sex body. The proportion of these cells in *Spo11*^*-/-*^ and in *Spo11*^*-/-*^ *p53*^*-/-*^ mice was indistinguishable (61.0% ± 3.5% vs. 63.0% ± 7.8%, respectively; p = 0.77, one-way ANOVA test; Fig 7C–7E). We conclude that absence of *p53* does not improve meiotic progression of *Spo11* mutant spermatocytes, thus supporting the conclusion that p53 family members are not involved in the arrest responding to sex body failure in mammals.

## Discussion

**Fig 8:**
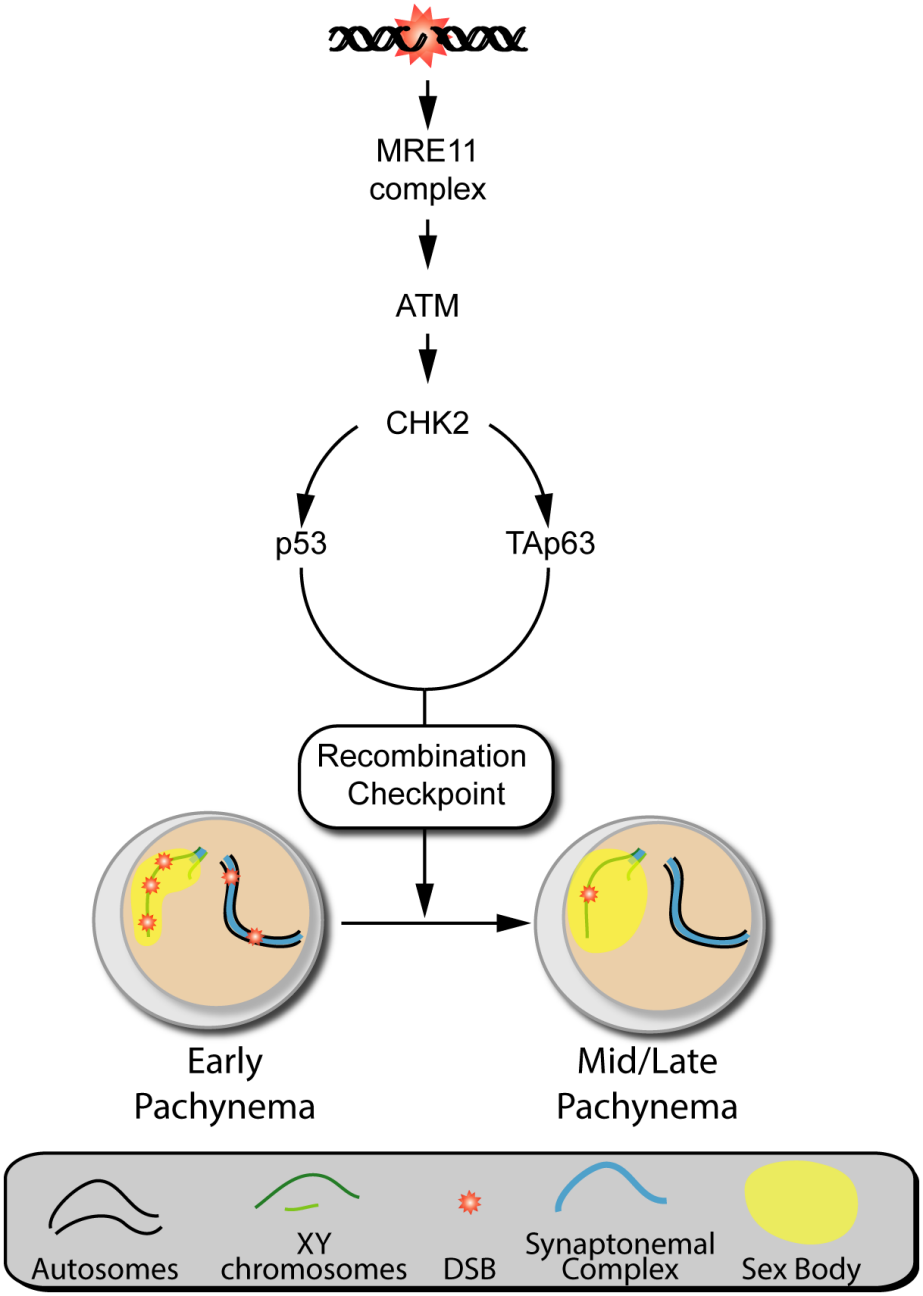
Signaling pathway leading to the activation of the recombination-dependent arrest. Based on the information presented in this and previous studies [36], we propose the following model of the signaling pathway required to activate the recombination-dependent arrest in mouse spermatocytes. DSBs are sensed by the MRE11 complex and lead to activatation of ATM, which in turn activates CHK2, leading to the upregulation of p53 and TAp63, which implement the recombination-dependent arrest that blocks progression to mid/late pachynema.

In this study, we provide new insights about the functionality of p53 family members in mouse meiotic surveillance mechanisms. Deficient recombination and/or sex body formation drives spermatocytes to arrest at pachynema and trigger programmed cell death, resulting in infertility. We previously reported that the MRN-ATM-CHK2 signaling pathway participates in the activation of recombination-dependent arrest in mouse spermatocytes (Fig 8 and [36]). Moreover, it was described that in females, the activation of CHK2, p53, and TAp63 is required to eliminate defective oocytes with persistent unrepaired DSBs [37]. Concordantly, results presented here show that p53 and TAp63 also participate in recombination-dependent arrest in spermatocytes (Fig 8). However, the third p53 family member, p73, does not participate in this arrest. By contrast, we report that p53 family members are not required for sex body formation dependent arrest.

Our data shed light on the importance of the p53 family in pachytene arrest in male mouse meiosis and resolve previous contradictory reports. An initial study observed that deletion of p53 partially rescued the phenotype of mutant spermatocytes that failed to repair DSBs, suggesting the involvement of p53 in recombination-dependent pachytene stage arrest [57]. However, two subsequent studies did not support this conclusion: one study analyzed the same mutants but could find no signs of rescue [58], while another study reported that p53 ablation had no impact on the meiotic progression of *Sycp3*^*-/-*^ spermatocytes, which fail to form proper chromosome axes and also accumulate unrepaired DSBs [59]. Thus, both later studies discounted a role for p53 in the so-called “pachytene checkpoint”. These discrepancies can be explained by developments in the intervening years, combined with results presented here. For instance, while the first study used H1t as a marker of meiotic progression (as we have done here), the subsequent studies did not. Furthermore, at the time these studies were conducted it was not known that there are two genetically independent mechanisms that trigger pachytene arrest in spermatocytes [29]. This is important because alleviating recombination-dependent arrest has no impact on surveillance of the sex body [36]. Consequently, cytological markers like H1t must be used to accurately define the arrest stage. Our data clearly provides evidence that the mammalian meiotic surveillance mechanism uses both p53 and TAp63 to activate recombination-dependent arrest at pachynema.

Our analysis also demonstrates that p73, despite being present in mouse spermatocytes, is dispensable for recombination-dependent arrest. A previous report proposed that p73 controls a p53-independent apoptotic response of mouse spermatogonia to irradiation [42]. Thus, it appears that, while p73 may be important to maintain genome integrity of premeiotic germ cells, p53 and TAp63 assume this role in spermatocytes.

Because *Trip13*^*mod/mod*^ *p53* and *Trip13*^*mod/mod*^ *TAp63* double mutant spermatocytes phenotypically resemble *Trip13*^*mod/mod*^ *Chk2*^*-/-*^ [36], we infer that p53 and TAp63 act downstream of CHK2 and that they are non-redundant for activation of recombination-dependent arrest. Furthermore, the observation of highly similar phenotypes in both *Trip13*^*mod/mod*^ *p53* and *Trip13*^*mod/mod*^ *TAp63* double mutants leads us to suggest that p53 and TAp63 are equivalently necessary to properly activate this arrest. p53 and TAp63 are intimately connected: they can promote transcription of the same genes [3] and can associate with each other through direct interaction [60].

There are several ways in which p53 and TAp63 could promote this arrest. For example, both proteins might form a heterotetramer that is required to activate the recombination-dependent response. Alternatively, p53 and TAp63 might act as homotetramers that work independently but additively to activate the same (or partially overlapping) set of target genes; in this model, activity of both is required in order to pass a certain response threshold. This latter option seems particularly appropriate for spermatocytes because they normally accumulate a certain level of unrepaired DSBs at pachynema. Specifically, the X chromosome accumulates DSBs [61] and because most of the X chromosome has no homologous partner, most of these DSBs remain unrepaired until late pachynema or later [62]. Thus, since all spermatocytes at pachynema could potentially activate this recombination-dependent arrest if it had a low threshold, it is plausible that requiring independent function of both p53 and TAp63 could make the threshold for arrest high enough to allow most wild-type spermatocyte progression. Interestingly, studies in yeast have established that sub-critical levels of DNA damage response are important for promoting proper recombination outcomes and coordinating meiotic progression with completion of recombination [63–65]. It is possible that regulation of TAp63 and/or p53 plays analogous roles in mouse meiosis. More studies will help us elucidate the nature of the interaction among p53 and TAp63 in order to control meiotic prophase progression.

Requirement for both p53 and TAp63 contrasts with what has been observed in females, where p53 and TAp63 appear to be substantially redundant. For example, whereas *Chk2* mutation completely rescued infertility in *Trip13* mutant females, deletion of *p53* only mildly increased the number of *Trip13*-deficient oocytes. Furthermore, absence of *TAp63* is required in addition to heterozygosity for *p53* in order to suppress the arrest observed in *Trip13* mutant oocytes [37].

These gender differences may reflect constraints tied to other sexual dimorphisms in mammalian gametogenesis. One key difference between the sexes is that meiosis in the testis is a continually renewed process that starts at the onset of sexual maturity and lasts through most or all of adulthood, whereas meiosis in the ovaries occurs in one wave during fetal development with oocytes then arresting at the end of meiotic prophase (around two days post-partum in mouse). Arrested oocytes do not resume meiosis until adulthood. These resting oocytes are also sensitive to DNA damage caused by ionizing radiation [37]. Importantly, it has been proposed that the same checkpoint mechanism that monitors meiotic DSB repair in fetal oocytes controls DNA integrity in the resting oocytes [37]. Thus, the fact that the recombination-dependent arrest machinery has to control genome integrity in resting oocytes may have resulted in a female-specific balance between p53 and TAp63 for responding to DNA damage during meiotic prophase.

Our observations that *Trip13* mutants present sex body defects resulting in inefficient MSCI and subsequent pachytene arrest corroborate our earlier conclusion that TRIP13 participates in the formation of the sex body [36]. Our results support a model whereby TRIP13 is required to properly load ATR onto unsynapsed X and Y axes to extend its signal to the chromatin, allowing proper H2AX phosphorylation and SUMO-1 loading. Additionally, because *Trip13*^*mod/mod*^ *p53*^*-/-*^, *Trip13*^*mod/mod*^ *TAp63*^*-/-*^, *Trip13*^*mod/mod*^ *p73*^*-/-*^, *Spo11*^*-/-*^ *p53*^*-/-*^ *and Spo11*^*-/-*^ *TAp63*^*-/-*^ double mutants all display arrest at epithelial stage IV, these results show that p53 family members are not required for sex body-deficient arrest. Therefore, these findings further strengthen the conclusion that the recombination-dependent and the sex body-deficient arrests are genetically separable mechanisms that respond to different offenses [36].

A next question would be to investigate components downstream of p53 and TAp63 in recombination-compromised spermatocytes. After induction of DNA damage in somatic cells, p53 induces transcription of *p21* [66] and pro-apoptotic genes such as *Bax* [67], *Puma* [68] and *Noxa* [69]. Remarkably, irradiated oocytes also require TAp63 to induce expression of *Puma* and *Noxa*, and irradiated *Puma*^*-/-*^ and *Puma*^*-/-*^ *Noxa*^*-/-*^ mice are protected from oocyte lost [70]. Therefore, future studies could be directed to determinate if this role of PUMA and NOXA, or other p53 family targets, is also conserved in DNA repair-compromised spermatocytes.

## Materials and methods

### Mutant mice

We used mice carrying *Trip13*, *p53*, *TAp63*, *p73*, *Spo11*, *Spo11 β*-only, *Dmc1 and Atm* mutations that were previously generated and described elsewhere [38,45,55,61,71–74]. All lines were maintained in a C57Bl/6-129/Sv mixed background. Experiments were performed using at least two animals (unless otherwise mentioned) and comparing them to wild-type littermates (when possible) or from animals from other litters from the same matings or closely related parents (See S3 Table for the relation between the control animals used in each experiment). Genotyping was performed by PCR analysis from tail extracted DNA as previously reported [38]. Experiments complied with U.S. and E.U. regulations and were approved by the Ethics Committee of the UAB and Catalan Government and by the MSKCC Institutional Animal Care and Use Committee.

### Histology and cytology

Testes from 2 to 4 months old adult mice were collected and processed for histology or cytology. For histology, testes were fixed overnight at 4°C with either Bouin’s solution or 4% paraformaldehyde, then embedded in paraffin and sectioned. For histological staging [49], testis sections fixed with Bouin’s solution were stained with Periodic Acid-Shiff (PAS) and Hematoxylin. Testis sections fixed with paraformaldehyde were used for apoptosis analysis. TUNEL staining was performed using the *In situ* cell death detection kit (Roche Diagnostics) following the manufacturer’s protocol. Immunostaining was performed after antigen retrieval protocols were applied on testis sections. Mouse monoclonal antibody against p53 (Cell signaling, 1:200) and a mouse monoclonal antibody against p63 (Abcam, 1:50) were used. The secondary antibody was raised in goat and conjugated with Cy3 (Jackson Immunoresearch Europe).

### Spermatocyte squashes, spreads and immunofluorescence

Squashed nuclear spermatocyte preparations, which preserve nuclear chromosome structure, were made as previously described [75]. Surface-spread nuclei were prepared as described previously [76]. Briefly, cell suspensions from frozen testes were minced in PBS, spermatocytes were treated with 1% lipsol and fixed with 0.15% Triton X-100 and 1% paraformaldehyde for two hours in a humid chamber at 4°C, air-dried, and washed with 0.4% Photo-flo (Kodak).

Before the staining, squashes or spreads were blocked with PTBG (0.2% BSA, 0.2% gelatin, 0.05% Tween-20 in PBS). Immunofluorescence staining was performed using standard methods [76]. Primary antibodies used were: mouse and rabbit anti-SYCP3 (Abcam, 1:400); mouse anti-γH2AX (Millipore, 1:400); guinea-pig anti-H1t (kind gift from M.A. Handel, Jackson Laboratory, 1:500); rabbit anti-RAD51 (Calbiochem, 1:100), rabbit anti-ATR (Calbiochem, 1:100) and mouse anti-SUMO-1 (Invitrogen, 1:100). Secondary antibodies used were all raised in goat and conjugated with FITC, Cy3 or Cy5 (Jackson Immunoresearch Europe). TUNEL staining was performed on immunofluorescent-stained slides as previously reported [36], and then slides were mounted with Vectashield (Vector Laboratories) reagent containing DAPI. Images were captured using a Zeiss Axioskop fluorescence microscope with a ProgRes C10 camera using ProgRes Pro 2.7.7 software and processed with Photoshop.

### RNA FISH and immunofluorescence

RNA FISH was performed with digoxigenin-labeled probes as described [36,53,77]. BAC DNA probes used for this study were: *Scml2*, RP24-204O18 (CHORI BACPAC library) and *Zfx*, bMQ-372M23 (Mouse bMQ BAC library). BAC-containing bacteria were grown in LB-chloramphenicol culture overnight at 37°C. A standard miniprep method was used to isolate BAC DNA. Approximately 2 μg of BAC DNA was labelled using DIG-Nick Translation Mix (Roche) and precipitated with Cot-1 DNA (Invitrogen) and salmon sperm DNA (Stratagene). Frozen testis cells were permeabilized with CSK buffer (100 mM NaCl, 300 mM sucrose, 3 mM MgCl_2_, 10 mM PIPES, 0.5% Triton X-100, 2 mM vanadyl ribonucleoside (New England Biolabs)), fixed with 4% paraformaldehyde, and dehydrated through an ice-cold ethanol series. DNA-BAC probes were denatured at 80°C, pre-hybridized at 37°C, and incubated with the sample overnight at 37°C. After stringency washes, digoxigenin was detected with anti-digoxigenin-FITC (1:10, Millipore). RNA FISH slides were then stained for immunofluorescence with anti-TOPBP1 (1:50, Abcam) and anti-γH2AX (1:100, Millipore). Samples were analyzed on an Olympus IX70 inverted microscope and computer-assisted (DeltaVision) CCD camera (Photometrics) was used to capture images (processed with ImageJ and Photoshop software).

### Statistical analysis

Student's t tests and one-way ANOVA tests were performed using GraphPad Prism software and/or GraphPad QuickCalcs online resource (http://www.graphpad.com/quickcalcs/). For comparing counts of γH2AX patches between genotypes at pachynema, we used t tests for simplicity since the data reasonably approximated a normal distribution. However, when we compared counts for diplotene cells, we used negative binomial regression because the count distributions were highly skewed for some genotypes and contained many zero values for all samples. Regression was calculated using the glm.nb function from the MASS package (version 7.3–33) in R (version 3.1.1).

## Supporting information

S1 Fig Absence of TAp63, but not p73, allows TRIP13-deficient spermatocytes to accumulate H1t despite having multiple unrepaired DSBs. Spermatocytes of the indicated stages and genotypes were immunostained for SYCP3 (green), H1t (blue), and γH2AX (red). The large γH2AX staining areas are the sex bodies (white arrows); the smaller γH2AX patches reflect unrepaired DSBs (orange arrowheads). Notice the presence of multiple unrepaired DSBs in *Trip13*^*mod/mod*^ *TAp63*^*-/-*^, but not in *Trip13*^*mod/mod*^ *p73*^*-/-*^. Scale bar in H represents 10 μm and applies to all panels.
(TIF)

S2 Fig γH2AX quantification. A representative spermatocyte stained for H1t, SYCP3, and ©H2AX is shown. Each counted γH2AX patch is shown as a yellow dot in panels C and D. Only those dots that touched the SC were scored. Scale bar in D represents 10 μm and applies to all panels.
(TIF)

S3 Fig *Trip13*^*mod/mod*^ *p53*^*-/-*^ spermatocytes with elevated numbers of RAD51 foci reach late pachynema and diplonema. Representative spermatocytes from the indicated genotypes are shown, stained for H1t (blue), SYCP3 (green), and RAD51 (red) (A and C). The image of the RAD51 channel alone is also provided (B and D). Scale bar in D represents 10 μm and applies to panels (A-D). (E) Quantification of RAD51 foci per spermatocyte. Horizontal lines represent means. Means (± SD) are indicated above the graph, and the number of cells counted (n) is indicated below. Number of animals analyzed per each genotype (N) is indicated in the key. Above the means, (n.s.) indicates p>0.05 (T test) and (*) indicates significantly different relative to *Trip13*^*mod/mod*^ (for Mid/Late pachynema, p = 0.0006, T test and for Diplonema, p = 0.00002, negative binomial regression).
(TIF)

S4 Fig Absence of TAp63, but not p73, in TRIP13-deficient spermatocytes drives them to apoptose at mid/late pachynema. Representative apoptotic spermatocytes from the *Trip13*^*mod/mod*^ *TAp63*^*-/-*^ (A–D) or *Trip13*^*mod/mod*^ *p73*^*-/-*^ (E–H) mutants were stained for SYCP3 and TUNEL (both in green), H1t (blue), and γH2AX (red). Scale bar in H represents 10 μm and applies to all panels.
(TIF)

S5 Fig Sex body defects and MSCI failure in TRIP13-deficient cells lacking TAp63. (A) *Trip13*^*mod/mod*^ *TAp63*^*-/-*^ pachytene spermatocyte stained for SYCP3 (green), H1t (blue), and γH2AX (red). Note the presence of an elongated sex body (arrow). (B) Representative *Trip13*^*mod/mod*^ *TAp63*^*-/-*^ pachytene spermatocyte stained for SYCP3 (green), ATR (red; note ATR signal displaying a discontinuous axis-constrained pattern, arrow), and DAPI (blue). (C) Representative *Trip13*^*mod/mod*^ *TAp63*^*-/-*^ pachytene spermatocyte stained for SYCP3 (green), SUMO-1 (red; showing a faint SUMO-1 sex body signal, arrow), and DAPI (blue). Scale bar in C represents 10 μm and applies to panels A–C. (D–I) Representative RNA-FISH performed on *Trip13*^*mod/mod*^ *TAp63*^*-/-*^ early pachytene spermatocytes, showing expression of *Scml2* (D–E) or *Zfx* (G–H) RNA signal (white, arrows). Cells were also stained for TOPBP1 (green), γH2AX (red), and DAPI (blue). Scale bar in I represents 10 μm and applies to panels D–H.
(TIF)

S6 Fig Mutation of *TAp63* or *p53* does not rescue *Spo11*^*-/-*^ testis size. Graph shows normalized testis weight (testis weight divided by body weight) of the indicated genotypes. The green shading includes wild type and the mutants that complete meiosis (*p53*^*-/-*^ or *TAp63*^*-/-*^). Yellow shading encompasses mutants that experience an arrest at metaphase of the first meiotic division (*Spo11*^*+/-*^ *Atm*^*-/-*^ and *Spo11*^*-/-*^ *tg-Spo11β*^*+/+*^). Pink shading indicates mutants that present pachytene arrest (*Spo11*^*-/-*^ or *Dmc1*^*-/-*^). Data from *Spo11*^*+/-*^ *Atm*^*-/-*^ were previously published [36]. Black horizontal lines represent the mean, which is also indicated above the corresponding genotype (mean ± SD). N show the number of animals analyzed for each genotype. Note that *Spo11*^*-/-*^ *p53*^*-/-*^ and *Spo11*^*-/-*^ *TAp63*^*-/-*^ double mutants have testis size comparable to *Spo11*^*-/-*^ mutants.
(TIF)

S1 Table TRIP13 mutants present defects in sex body formation. (DOCX)

S2 Table Expression of *Scml2* and *Zfx* on early pachytene cells from wild type and mutant mice. (DOCX)

S3 Table Relation of animals used in this study. (DOCX)
