# Supplementary figures and images for: p53 and TAp63 participate in the recombination-dependent pachytene arrest in mouse spermatocytes
Source: PLoS Genet. 2017 Jun 15;13(6):e1006845. doi: 10.1371/journal.pgen.1006845 (PMC5491309; doi:10.1371/journal.pgen.1006845)

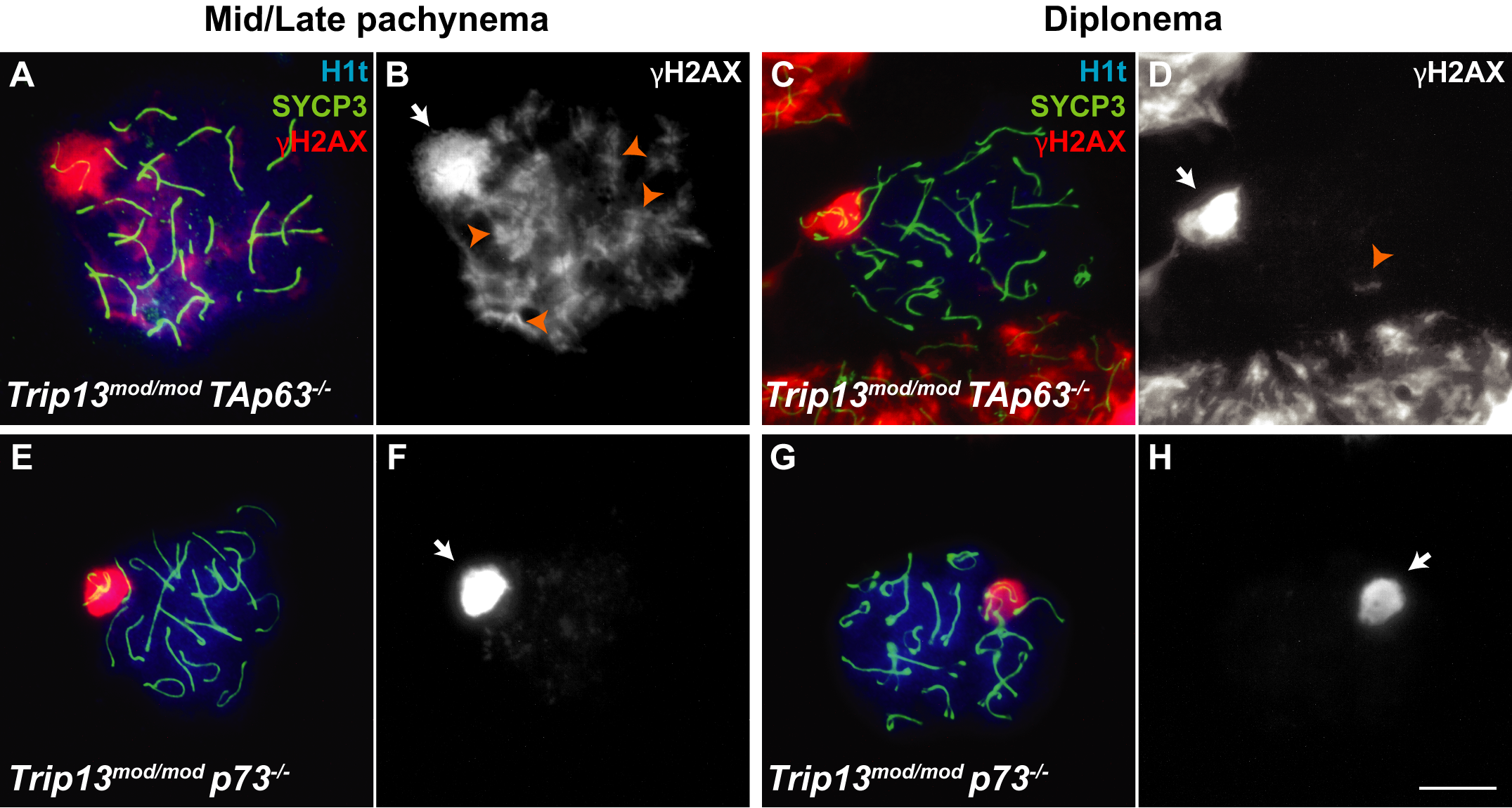

Supplement: S1 Fig — Spermatocytes of the indicated stages and genotypes were immunostained for SYCP3 (green), H1t (blue), and γH2AX (red). The large γH2AX staining areas are the sex bodies (white arrows); the smaller γH2AX patches reflect unrepaired DSBs (orange arrowheads). Notice the presence of multiple unrepaired DSBs in Trip13mod/mod TAp63-/-, but not in Trip13mod/mod p73-/-. Scale bar in H represents 10 μm and applies to all panels. (TIF) [file pgen.1006845.s001.tif]

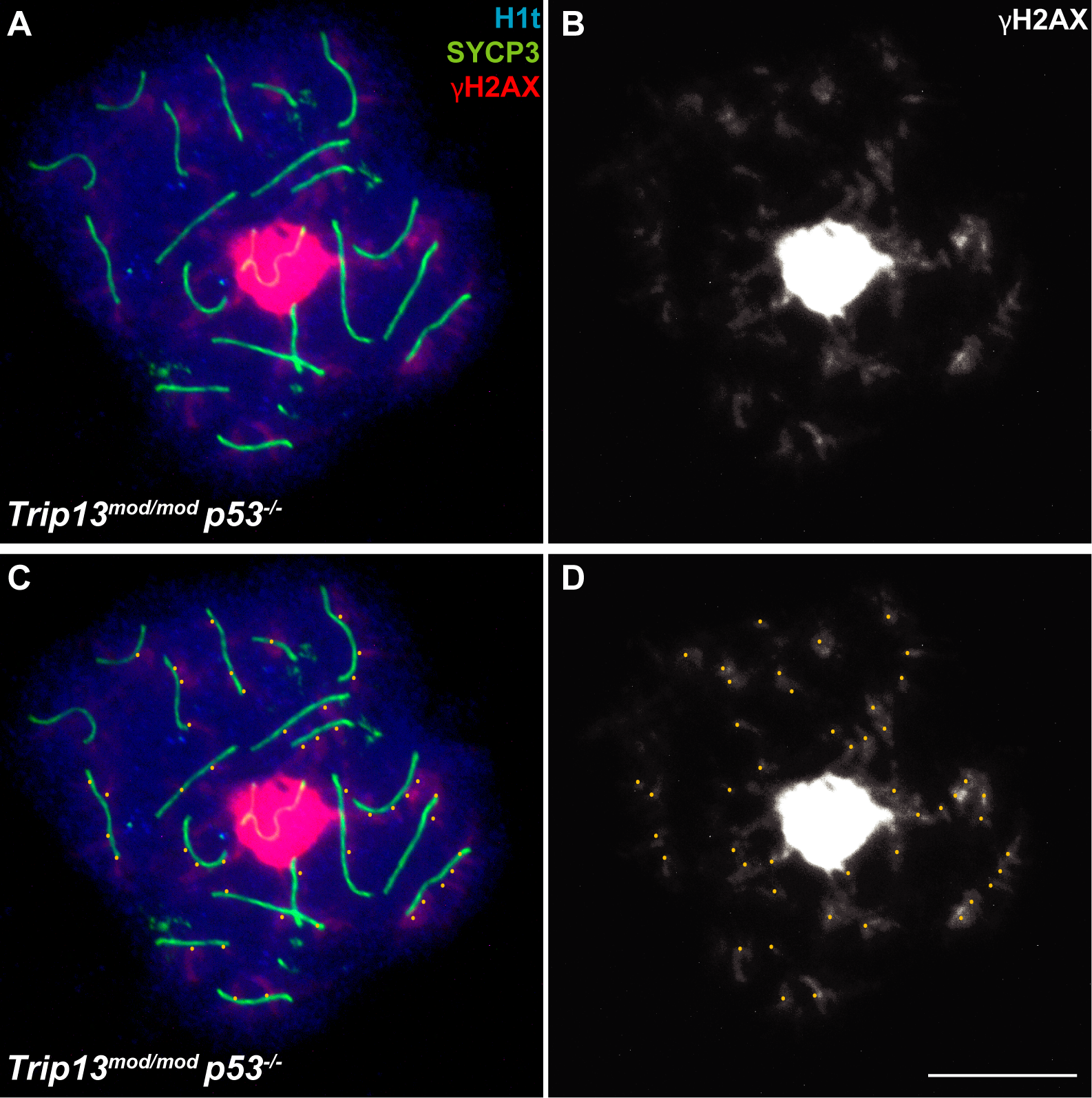

Supplement: S2 Fig — A representative spermatocyte stained for H1t, SYCP3, and ©H2AX is shown. Each counted γH2AX patch is shown as a yellow dot in panels C and D. Only those dots that touched the SC were scored. Scale bar in D represents 10 μm and applies to all panels. (TIF) [file pgen.1006845.s002.tif]

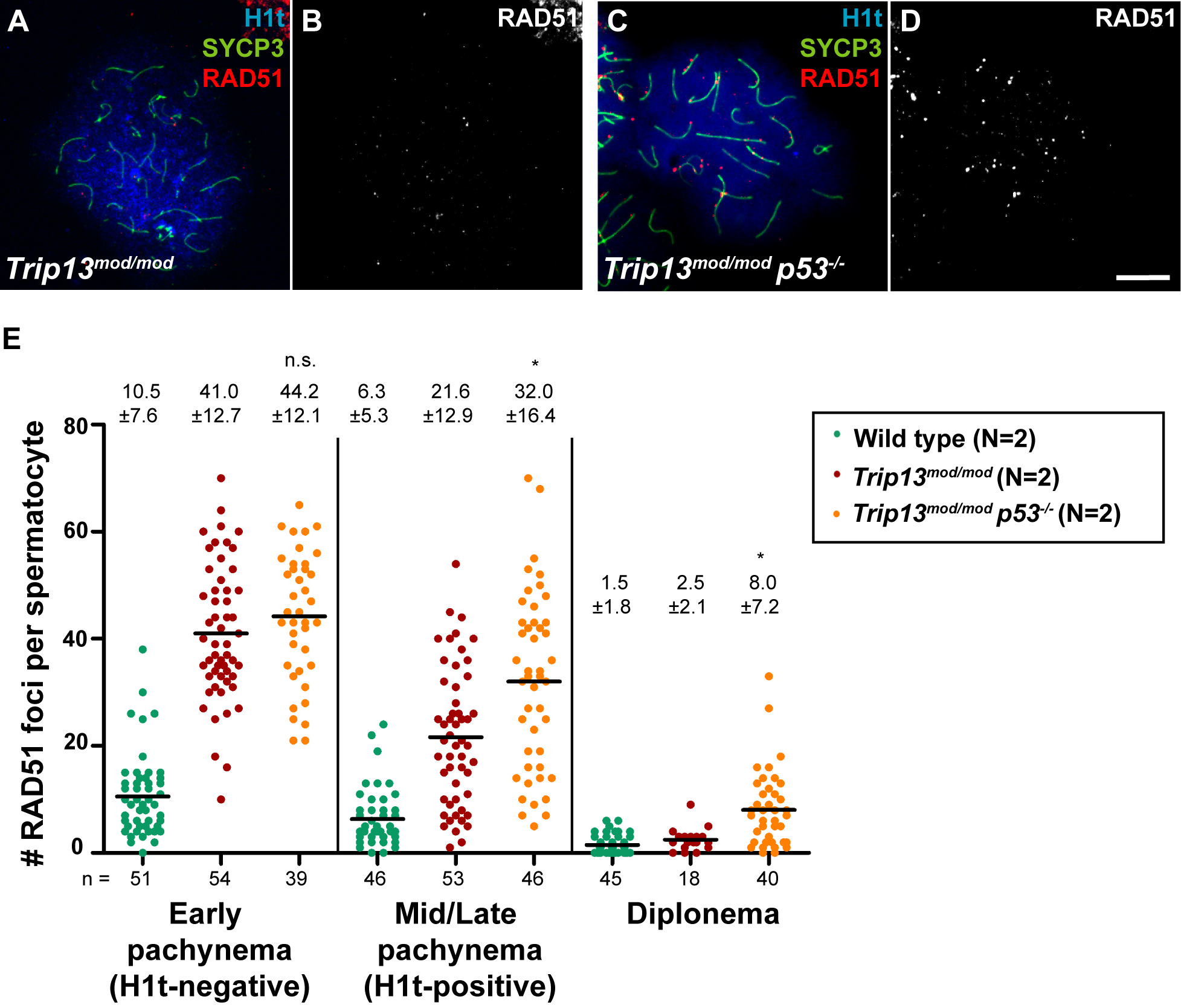

Supplement: S3 Fig — Representative spermatocytes from the indicated genotypes are shown, stained for H1t (blue), SYCP3 (green), and RAD51 (red) (A and C). The image of the RAD51 channel alone is also provided (B and D). Scale bar in D represents 10 μm and applies to panels (A-D). (E) Quantification of RAD51 foci per spermatocyte. Horizontal lines represent means. Means (± SD) are indicated above the graph, and the number of cells counted (n) is indicated below. Number of animals analyzed per each genotype (N) is indicated in the key. Above the means, (n.s.) indicates p>0.05 (T test) and (*) indicates significantly different relative to Trip13mod/mod (for Mid/Late pachynema, p = 0.0006, T test and for Diplonema, p = 0.00002, negative binomial regression). (TIF) [file pgen.1006845.s003.tif]

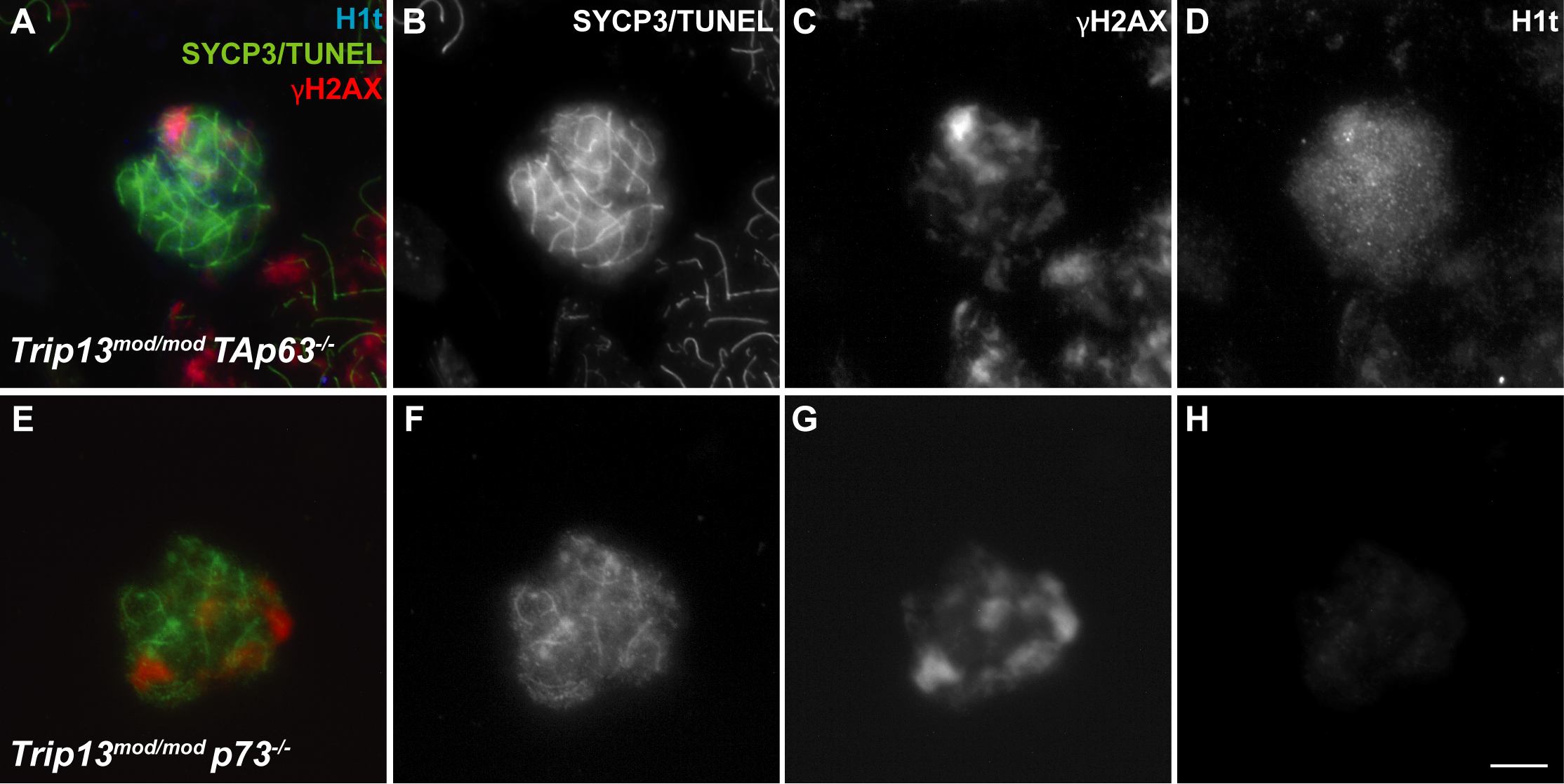

Supplement: S4 Fig — Representative apoptotic spermatocytes from the Trip13mod/mod TAp63-/- (A–D) or Trip13mod/mod p73-/- (E–H) mutants were stained for SYCP3 and TUNEL (both in green), H1t (blue), and γH2AX (red). Scale bar in H represents 10 μm and applies to all panels. (TIF) [file pgen.1006845.s004.tif]

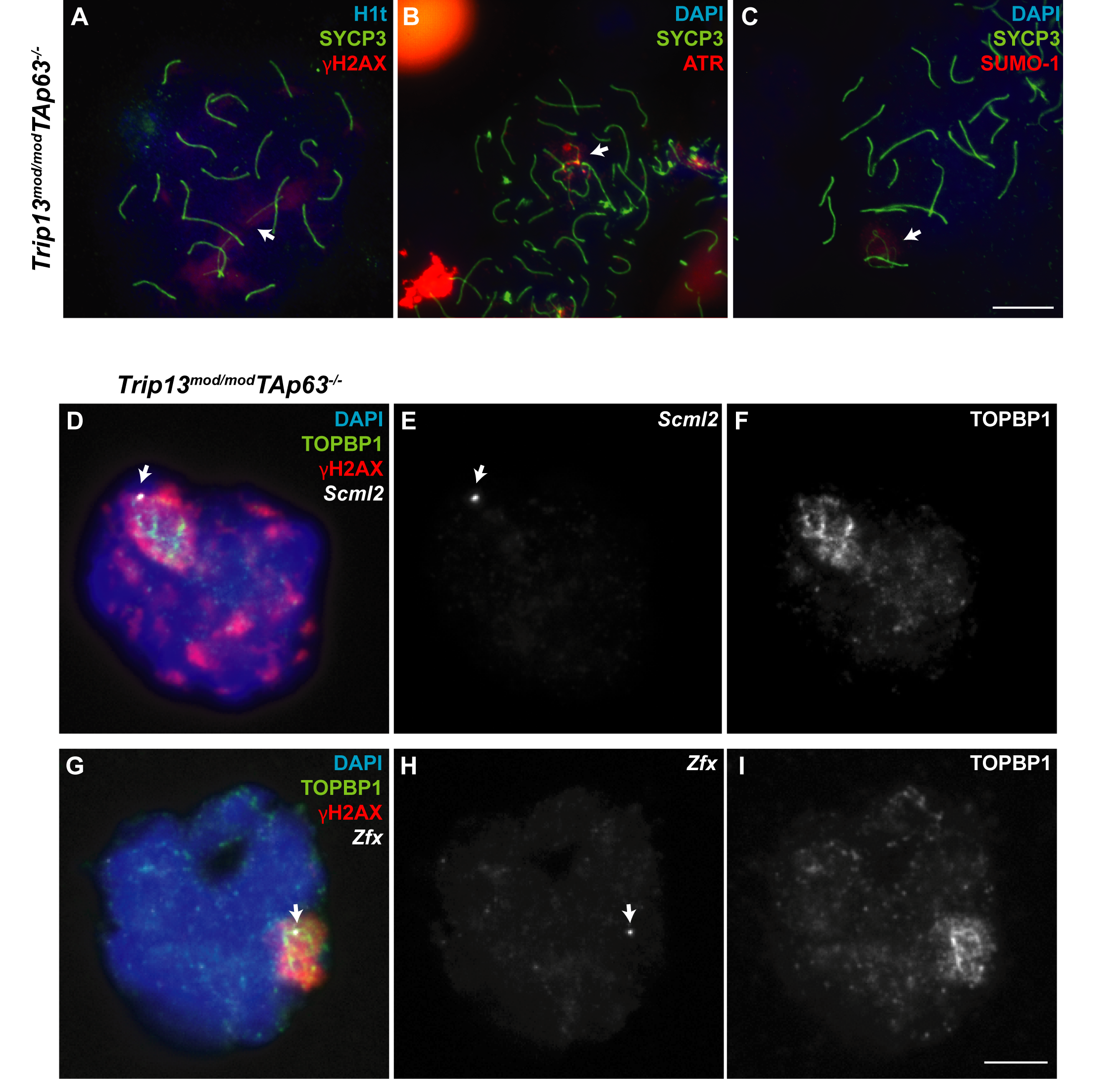

Supplement: S5 Fig — (A) Trip13mod/mod TAp63-/- pachytene spermatocyte stained for SYCP3 (green), H1t (blue), and γH2AX (red). Note the presence of an elongated sex body (arrow). (B) Representative Trip13mod/mod TAp63-/- pachytene spermatocyte stained for SYCP3 (green), ATR (red; note ATR signal displaying a discontinuous axis-constrained pattern, arrow), and DAPI (blue). (C) Representative Trip13mod/mod TAp63-/- pachytene spermatocyte stained for SYCP3 (green), SUMO-1 (red; showing a faint SUMO-1 sex body signal, arrow), and DAPI (blue). Scale bar in C represents 10 μm and applies to panels A–C. (D–I) Representative RNA-FISH performed on Trip13mod/mod TAp63-/- early pachytene spermatocytes, showing expression of Scml2 (D–E) or Zfx (G–H) RNA signal (white, arrows). Cells were also stained for TOPBP1 (green), γH2AX (red), and DAPI (blue). Scale bar in I represents 10 μm and applies to panels D–H. (TIF) [file pgen.1006845.s005.tif]

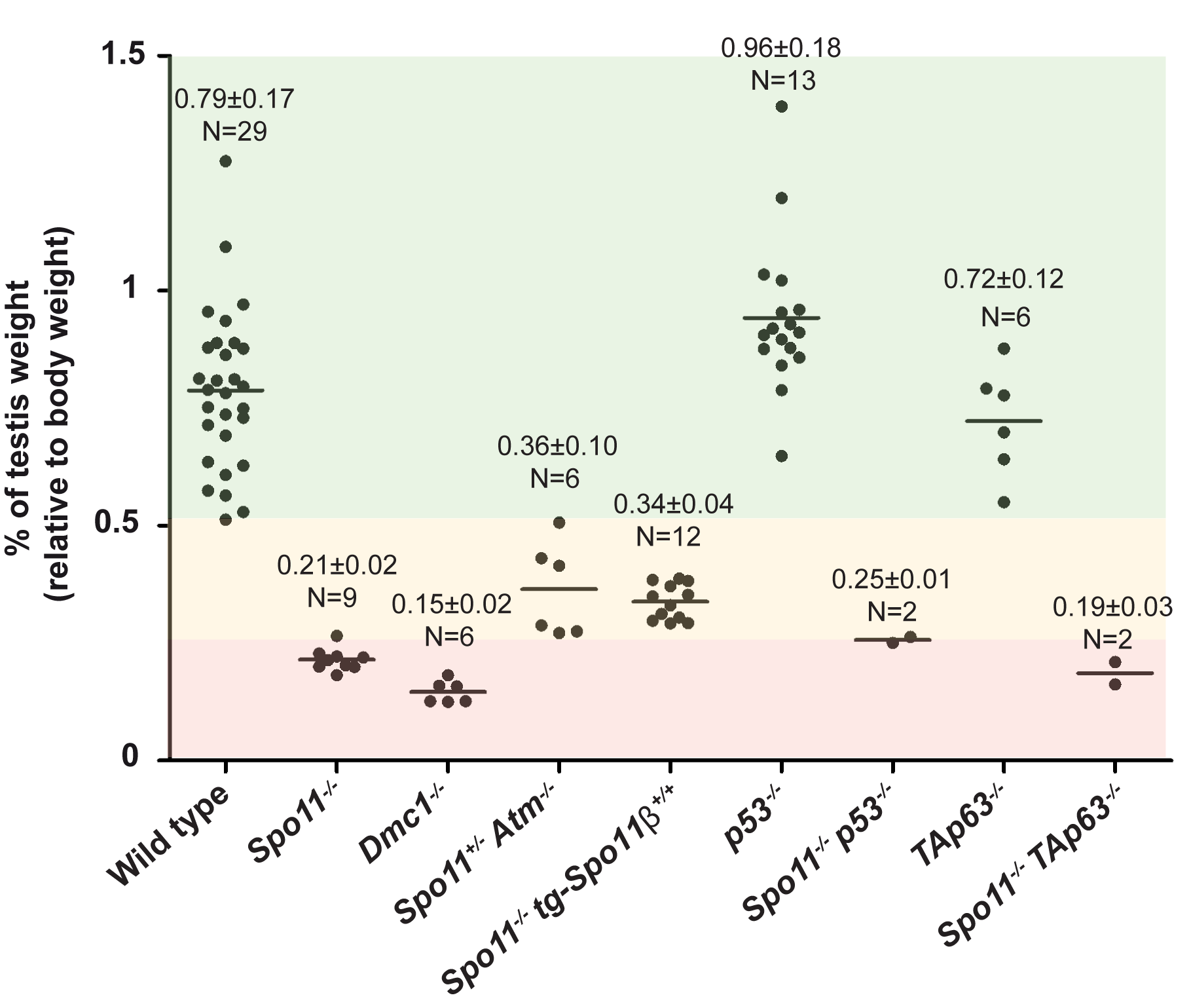

Supplement: S6 Fig — Graph shows normalized testis weight (testis weight divided by body weight) of the indicated genotypes. The green shading includes wild type and the mutants that complete meiosis (p53-/- or TAp63-/-). Yellow shading encompasses mutants that experience an arrest at metaphase of the first meiotic division (Spo11+/- Atm-/- and Spo11-/- tg-Spo11β+/+). Pink shading indicates mutants that present pachytene arrest (Spo11-/- or Dmc1-/-). Data from Spo11+/- Atm-/- were previously published [36]. Black horizontal lines represent the mean, which is also indicated above the corresponding genotype (mean ± SD). N show the number of animals analyzed for each genotype. Note that Spo11-/- p53-/- and Spo11-/- TAp63-/- double mutants have testis size comparable to Spo11-/- mutants. (TIF) [file pgen.1006845.s006.tif]
