# Supplementary material for: p53 and TAp63 participate in the recombination-dependent pachytene arrest in mouse spermatocytes
Source: PLoS Genet. 2017 Jun 15;13(6):e1006845. doi: 10.1371/journal.pgen.1006845 (PMC5491309; doi:10.1371/journal.pgen.1006845)
Supplement: S1 Table — (DOCX) [file pgen.1006845.s007.docx]

|  | **Wild type** | ***Trip13^mod/mod^*** | ***Trip13^mod/mod^ p53^-/-^*** | ***Trip13^mod/mod^ TAp63^-/-^*** |
| --- | --- | --- | --- | --- |
| **γH2AX** |  |  |  |  |
| Elongated sex body | 16.7±7.6 % | 61.7±12.6 % | 71.7±5.8 % | 68.3±2.9 % |
| Round sex body | 83.3±7.6 % | 38.3±12.6 % | 28.3±5.8 % | 31.7±2.9 % |
| (n cells) | 60 | 60 | 60 | 60 |
| (n animals) | 3 | 3 | 3 | 3 |
| **ATR** |  |  |  |  |
| No staining | 0.0±0.0 % | 4.3±4.3 % | 2.7±2.3 % | 9.0±2.6 % |
| Discontinuous XY axis staining | 6.4±3.1 % | 51.3±7.3 % | 64.7±9.0 % | 53.0±3.6 % |
| Complete XY axis staining | 25.2±3.3 % | 29.0±5.5 % | 24.3±6.0 % | 22.3±4.7 % |
| Chromatin staining | 68.4±3.0 % | 15.5±8.3 % | 9.3±2.1 % | 16.0±2.6 % |
| (n cells) | 500 | 400 | 300 | 300 |
| (n animals) | 5 | 4 | 3 | 3 |
| **SUMO-1** |  |  |  |  |
| No staining | 1.4±1.1 % | 44.2±16.4 % | 27.3±15.9 % | 48.0±4.4 % |
| Faint staining | 13.8±4.0 % | 41.0±12.6 % | 51.0±10.5 % | 40.0±6.1 % |
| Strong staining | 84.8±4.8 % | 14.8±7.9 % | 21.7±5.8 % | 12.0±5.3 % |
| (n cells) | 500 | 500 | 300 | 300 |
| (n animals) | 5 | 5 | 3 | 3 |
