# Supplementary material for: p53 and TAp63 participate in the recombination-dependent pachytene arrest in mouse spermatocytes
Source: PLoS Genet. 2017 Jun 15;13(6):e1006845. doi: 10.1371/journal.pgen.1006845 (PMC5491309; doi:10.1371/journal.pgen.1006845)
Supplement: S2 Table — (DOCX) [file pgen.1006845.s008.docx]

|  | Wild type | | *Trip13^mod/mod^* | *Trip13^mod/mod^ p53^-/-^* | *Trip13^mod/mod^ TAp63^-/-^* |
| --- | --- | --- | --- | --- | --- |
| % of early pachytene cells expressing *Scml2* (mean ± SD) | 17.1 ±1.9 | 27.5 ±0.0* | | 28.8** | 34.4 ±4.4* |
| Cells analyzed | 200 | 160 | | 80 | 160 |
| Mice analyzed | 4 | 2 | | 1 | 2 |
| % of early pachytene cells expressing *Zfx* (mean ± SD) | 10.9 ±3.0 | 27.2 ±3.2* | | 33.8** | 30.6 ±0.9* |
| Cells analyzed | 225 | 158 | | 80 | 160 |
| Mice analyzed | 4 | 2 | | 1 | 2 |
| *  ** | Significantly different from wild type, P < 0.05 one-way ANOVA and Tukey's multiple comparison test.  Significantly different from wild type, P < 0.05 Fisher’s exact test. | | | | |
