# Supplementary material for: p53 and TAp63 participate in the recombination-dependent pachytene arrest in mouse spermatocytes
Source: PLoS Genet. 2017 Jun 15;13(6):e1006845. doi: 10.1371/journal.pgen.1006845 (PMC5491309; doi:10.1371/journal.pgen.1006845)
Supplement: S3 Table — (DOCX) [file pgen.1006845.s009.docx]

| **Experiment** | **Genotype** | **Animal Code** | **Complete Genotype** |
| --- | --- | --- | --- |
| Percentage of H1t-positive spermatocytes (Fig 1B) | Wild type | 2 | *Trip13+/+* |
|  |  | 4 | *Trip13+/+* |
|  |  | 5 | *Trip13+/+* |
|  |  | 6 | *Trip13+/+ p53+/+* |
|  | Trip13 | 1 | *Trip13mod/mod* |
|  |  | 5 | *Trip13mod/mod* |
|  |  | 6 | *Trip13mod/mod* |
|  |  | 7 | *Trip13mod/mod p53+/+* |
|  | Trip13 p53 | 1 | *Trip13mod/mod p53-/-* |
|  |  | 2 | *Trip13mod/mod p53-/-* |
|  |  | 3 | *Trip13mod/mod p53-/-* |
|  |  | 4 | *Trip13mod/mod p53-/-* |
|  | Trip13 TAp63 | 2 | *Trip13mod/mod TAp63-/-* |
|  |  | 3 | *Trip13mod/mod TAp63-/-* |
|  |  | 4 | *Trip13mod/mod TAp63-/-* |
|  | Trip13 p73 | 1 | *Trip13mod/mod p73-/-* |
|  |  | 2 | *Trip13mod/mod p73-/-* |
| Number of gH2AX patches per spermatocyte (Fig 1C-K) | Wild type | 1 | *Trip13+/+ p53+/+* |
|  |  | 2 | *Trip13+/+* |
|  |  | 3 | *Trip13+/+* |
|  | Trip13 | 1 | *Trip13mod/mod p53+/+* |
|  |  | 2 | *Trip13mod/mod p53+/+* |
|  |  | 3 | *Trip13mod/mod TAp63+/+* |
|  |  | 4 | *Trip13mod/mod TAp63+/+* |
|  | Trip13 p53 | 1 | *Trip13mod/mod p53-/-* |
|  |  | 2 | *Trip13mod/mod p53-/-* |
|  |  | 3 | *Trip13mod/mod p53-/-* |
|  | Trip13 TAp63 | 1 | *Trip13mod/mod TAp63-/-* |
|  |  | 2 | *Trip13mod/mod TAp63-/-* |
|  |  | 3 | *Trip13mod/mod TAp63-/-* |
|  | Trip13 p73 | 1 | *Trip13mod/mod p73-/-* |
|  |  | 2 | *Trip13mod/mod p73-/-* |
| Percentage of apoptotic spermatocytes (Fig 2) | Trip13 | 1 | *Trip13mod/mod p53+/+* |
|  |  | 2 | *Trip13mod/mod p53+/+* |
|  |  | 3 | *Trip13mod/mod TAp63+/+* |
|  |  | 4 | *Trip13mod/mod TAp63+/+* |
|  | Trip13 p53 | 1 | *Trip13mod/mod p53-/-* |
|  |  | 2 | *Trip13mod/mod p53-/-* |
|  |  | 3 | *Trip13mod/mod p53-/-* |
|  | Trip13 TAp63 | 1 | *Trip13mod/mod TAp63-/-* |
|  |  | 2 | *Trip13mod/mod TAp63-/-* |
|  |  | 3 | *Trip13mod/mod TAp63-/-* |
|  | Trip13 p73 | 1 | *Trip13mod/mod p73-/-* |
|  |  | 2 | *Trip13mod/mod p73-/-* |
| Apoptotic cells per tubule (Fig 3A-D) | wild type | 7 | *Trip13+/+ p53+/+* |
|  |  | 8 | *Trip13+/+ p53+/+* |
|  |  | 9 | *Trip13+/+ p53+/+* |
|  | Trip13 | 1 | *Trip13mod/mod p53+/+* |
|  |  | 8 | *Trip13mod/mod* |
|  |  | 9 | *Trip13mod/mod p53+/+* |
|  | Trip13 p53 | 1 | *Trip13mod/mod p53-/-* |
|  |  | 2 | *Trip13mod/mod p53-/-* |
|  |  | 4 | *Trip13mod/mod p53-/-* |
|  | Trip13 TAp63 | 2 | *Trip13mod/mod TAp63-/-* |
|  |  | 3 | *Trip13mod/mod TAp63-/-* |
|  |  | 4 | *Trip13mod/mod TAp63-/-* |
| Histology (Fig 3E-I and Fig 5) | Wild type | 10 | Trip13+/+ TAp63+/+ |
|  |  | 13 | Trip13+/+ p53+/+ |
|  |  | 17 | Trip13+/+ p53+/+ |
|  | Trip13 | 9 | Trip13mod/mod p53+/+ |
|  |  | 10 | Trip13mod/mod p53+/+ |
|  |  | 13 | Trip13mod/mod p53+/+ |
|  | Trip13 p53 | 1 | Trip13mod/mod p53-/- |
|  |  | 2 | Trip13mod/mod p53-/- |
|  | Trip13 TAp63 | 2 | Trip13mod/mod TAp63-/- |
|  |  | 4 | Trip13mod/mod TAp63-/- |
|  | Trip13 p73 | 1 | Trip13mod/mod p73-/- |
|  | Spo11 | 1 | Spo11-/- |
|  |  | 4 | Spo11-/- |
|  | Spo11 p53 | 1 | Spo11-/- p53-/- |
|  |  | 2 | Spo11-/- p53-/- |
| ATR, SUMO1 staining (Fig 4D-I and Sup Fig 5) | Wild type | 2 | *Trip13+/+* |
|  |  | 9 | *Trip13+/+ p53+/+* |
|  |  | 11 | *Trip13+/+* |
|  |  | 12 | *Trip13+/+ p53+/+* |
|  | Trip13 | 1 | *Trip13mod/mod p53+/+* |
|  |  | 2 | *Trip13mod/mod p53+/+* |
|  |  | 3 | *Trip13mod/mod TAp63+/+* |
|  |  | 4 | *Trip13mod/mod TAp63+/+* |
|  | Trip13 p53 | 1 | *Trip13mod/mod p53-/-* |
|  |  | 2 | *Trip13mod/mod p53-/-* |
|  |  | 3 | *Trip13mod/mod p53-/-* |
|  | Trip13 TAp63 | 1 | *Trip13mod/mod TAp63-/-* |
|  |  | 2 | *Trip13mod/mod TAp63-/-* |
|  |  | 3 | *Trip13mod/mod TAp63-/-* |
| gH2AX sex body staining (Fig 4A-C and Sup Fig 5) | Wild type | 1 | *Trip13+/+ p53+/+* |
|  |  | 2 | *Trip13+/+* |
|  |  | 3 | *Trip13+/+* |
|  | Trip13 | 2 | *Trip13mod/mod p53+/+* |
|  |  | 3 | *Trip13mod/mod TAp63+/+* |
|  |  | 4 | *Trip13mod/mod TAp63+/+* |
|  | Trip13 p53 | 1 | *Trip13mod/mod p53-/-* |
|  |  | 2 | *Trip13mod/mod p53-/-* |
|  |  | 3 | *Trip13mod/mod p53-/-* |
|  | Trip13 TAp63 | 1 | *Trip13mod/mod TAp63-/-* |
|  |  | 2 | *Trip13mod/mod TAp63-/-* |
|  |  | 3 | *Trip13mod/mod TAp63-/-* |
| RAD51 counts (Sup Fig 3) | Wild type | 1 | *Trip13+/+ p53+/+* |
|  |  | 13 | *Trip13+/+ p53+/+* |
|  | Trip13 | 2 | *Trip13mod/mod p53+/+* |
|  |  | 9 | *Trip13mod/mod p53+/+* |
|  | Trip13 p53 | 1 | *Trip13mod/mod p53-/-* |
|  |  | 2 | *Trip13mod/mod p53-/-* |
| Spo11 p53 cytology (Fig 5) | Spo11 | 2 | *Spo11-/-* |
|  |  | 3 | *Spo11-/-* |
|  | Spo11 p53 | 1 | *Spo11-/- p53-/-* |
|  |  | 2 | *Spo11-/- p53-/-* |
| RNA FISH (Fig 4J-K and Sup Fig 5) | Wild type | 14 | *Trip13+/+* |
|  |  | 15 | *Trip13+/+* |
|  |  | 16 | *Trip13+/+ p53+/+* |
|  | Trip13 | 11 | *Trip13mod/mod p53+/+* |
|  |  | 12 | *Trip13mod/mod p53+/-* |
|  | Trip13 p53 | 1 | *Trip13mod/mod p53-/-* |
|  | Trip13 TAp63 | 1 | *Trip13mod/mod TAp63-/-* |
|  |  | 4 | *Trip13mod/mod TAp63-/-* |
| p63 staining | Wild type | 17 | *Trip13+/+ p53+/+* |
|  |  | 13 | *Trip13+/+ p53+/+* |
|  | Trip13 | 9 | *Trip13mod/mod p53+/+* |
|  |  | 13 | *Trip13mod/mod p53+/+* |
|  | Trip13 p53 | 2 | *Trip13mod/mod p53-/-* |
|  |  | 1 | *Trip13mod/mod p53-/-* |
| p53 staining | Wild type | 17 | *Trip13+/+ p53+/+* |
|  |  | 13 | *Trip13+/+ p53+/+* |
|  | Trip13 | 9 | *Trip13mod/mod p53+/+* |
|  |  | 13 | *Trip13mod/mod p53+/+* |
|  | Trip13 TAp63 | 2 | *Trip13mod/mod TAp63-/-* |
|  |  | 4 | *Trip13mod/mod TAp63-/-* |
